# Tailoring glioblastoma treatment based on longitudinal analysis of post-surgical tumor microenvironment

**DOI:** 10.1186/s13046-024-03231-4

**Published:** 2024-11-28

**Authors:** Chiara Bastiancich, Emmanuel Snacel-Fazy, Samantha Fernandez, Stéphane Robert, Roberta Stacchini, Léa Plantureux, Sébastien Boissonneau, Benoit Testud, Benjamin Guillet, Franck Debarbieux, Hervé Luche, Dominique Figarella-Branger, Marie-Anne Estève, Emeline Tabouret, Aurélie Tchoghandjian

**Affiliations:** 1 Aix-Marseille Univ, CNRS, INP, Institute of Neurophysiopathology UMR7051, Team Gliomagenesis and Microenvironment, Faculté des Sciences Médicales et Paramédicales – Secteur Timone, 27, Bd Jean Moulin, Marseille, 13005 France; 2 Department of Drug Science and Technology, University of Turin, Turin, 10125 Italy; 3 UCLouvain, Louvain Drug Research Institute, Advanced Drug Delivery and Biomaterials, Avenue Mounier 73, Brussels, 1200 Belgium; 4 Aix-Marseille Univ, Réseau Préclinique Et Translationnel de Recherche en Neuro-Oncologie, Plateforme PETRA“TECH”, Marseille, 13005 France; 5 Aix Marseille Univ, CNRS, CERIMED, Marseille, France; 6 Aix Marseille Univ, INSERM, INRAE, C2VN Marseille, France; 7 Department of Neuro-Surgery, AP-HM, Hôpital Universitaire Timone, Marseille, 13005 France; 8 Department of Neuro-Surgery, Valenciennes Hospital, Valenciennes, 59300 France; 9 Aix Marseille Univ, CNRS, CRMBM, Marseille, France; 10 Aix Marseille Univ, APHM, Hôpital Universitaire Timone, CEMEREM, Marseille, 13005 France; 11 Department of Neuroradiology, Aix Marseille Univ, APHM, Hôpital Universitaire Timone, Marseille, 13005 France; 12 Aix Marseille Univ, APHM, Hôpital Timone, Pôle Pharmacie, Radiopharmacie, Marseille, 13005 France; 13 Aix Marseille Univ, CNRS, INT, Inst Neurosci Timone, Marseille, France; 14 Institut Universitaire de France, Paris, 75005 France; 15 Aix-Marseille Univ, CNRS, INSERM, CIPHE, Marseille, 13009 France; 16 Aix Marseille Univ, APHM, Hôpital Timone, Service Pharmacie, Marseille, 13005 France; 17 AP-HM, CHU Timone, Service de Neurooncologie, Marseille, France; 18 Aix-Marseille Univ, Réseau Préclinique Et Translationnel de Recherche en Neuro-Oncologie, Plateforme PE“TRANSLA”, Marseille, 13005 France

**Keywords:** Brain tumor surgery, Neuro-oncology, Glioblastoma, Tumor microenvironment, Drug Delivery, Targeted therapy

## Abstract

Glioblastoma (GBM), an incurable primary brain tumor, typically requires surgical intervention followed by chemoradiation; however, recurrences remain fatal. Our previous work demonstrated that a nanomedicine hydrogel (GemC_12_-LNC) delays recurrence when administered post-surgery. However, tumor debulking also triggers time-dependent immune reactions that promote recurrence at the resection cavity borders. We hypothesized that combining the hydrogel with an immunomodulatory drug could enhance therapeutic outcomes. A thorough characterization of the post-surgical microenvironment (SMe) is crucial to guide combinatorial approaches.

In this study, we performed cellular resolution imaging, flow cytometry and spatial hyperplexed immunofluorescence imaging to characterize the SMe in a syngeneic mouse model of tumor resection. Owing to our dynamic approach, we observed transient opening of the blood–brain barrier (BBB) during the first week after surgery. BBB permeability post-surgery was also confirmed in GBM patients. In our murine model, we also observed changes in immune cell morphology and spatial location post-surgery over time in resected animals as well as the accumulation of reactive microglia and anti-inflammatory macrophages in recurrences compared to unresected tumors since the first steps of recurrence growth. Therefore we investigated whether starting a systemic treatment with the SMAC mimetic small molecule (GDC-0152) directly after surgery would be beneficial for enhancing microglial anti-tumoral activity and decreasing the number of anti-inflammatory macrophages around the GemC_12_-LNC hydrogel-loaded tumor cavity. The immunomodulatory effects of this drug combination was firstly shown in patient-derived tumoroids. Its efficacy was confirmed in vivo by survival analysis and correlated with reversal of the immune profile as well as delayed tumor recurrence.

This comprehensive study identified critical time frames and immune cellular targets within the SMe, aiding in the rational design of combination therapies to delay recurrence onset. Our findings suggest that post-surgical systemic injection of GDC-0152 in combination with GemC_12_-LNC local treatment is a promising and innovative approach for managing GBM recurrence, with potential for future translation to human patient.

**Graphical Abstract:**

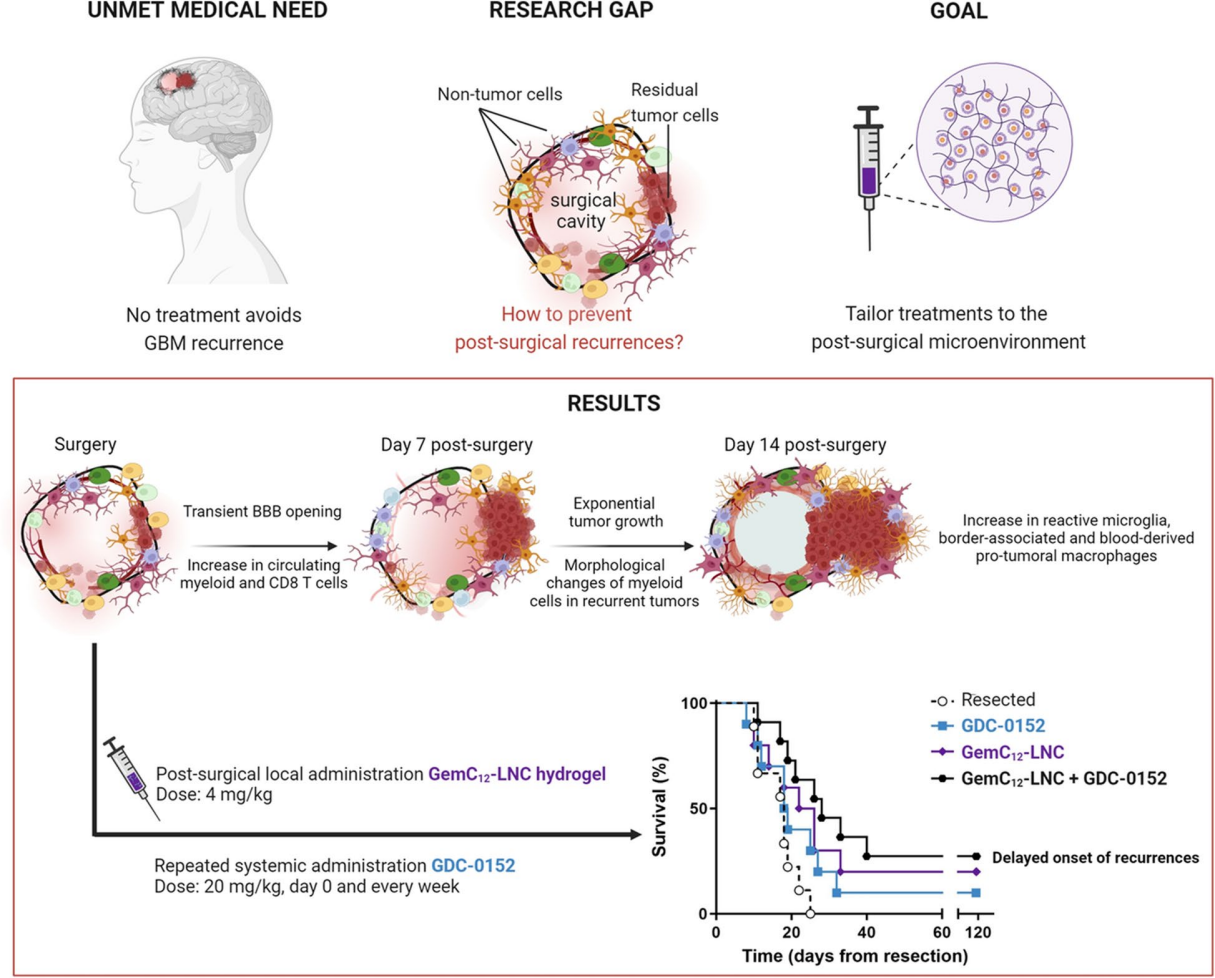

**Supplementary Information:**

The online version contains supplementary material available at 10.1186/s13046-024-03231-4.

## Introduction

Glioblastoma (GBM) is the most common and aggressive primary brain tumor in adults. Its fast and unpredictable appearance, lack of effective treatments and poor prognosis (5-year survival rate < 10%) make GBM an unsolved medical challenge [1]. The standard of care therapy for GBM patients includes surgery of the accessible tumor followed, several weeks later, by radiotherapy and concomitant adjuvant oral chemotherapy with temozolomide [2, 3]. Despite the widest range of tumor resection, residual tumor cells are always present post-surgery. They can be stimulated by post-operative healing processes, leading to a vicious cycle of inflammation, angiogenesis, and tumor regrowth and promoting local recurrences [4, 5]. These recurrences mainly arise along the post-surgical cavity borders and no resolutive treatment succeeds in inhibiting their onset and proliferation [6, 7]. Local administration of chemotherapeutic agents in the resection cavity at the end of surgery appears promising for inhibiting recurrence. This approach enables sustained release of therapeutic concentrations near residual tumor cells, inhibiting their proliferation while minimizing systemic side effects [8]. However, since the approval of the carmustine-loaded wafer Gliadel^®^ in 1996 — which is rarely used in clinics — no other local GBM treatment has received regulatory approval [8–10]. We previously demonstrated that delivering hydrogels loaded with nanomedicines in the post-surgical cavity of GBM is a safe and promising therapeutic approach to kill residual tumor cells in the gap between surgery and chemoradiation [11–16]. For example, the lauroyl-gemcitabine lipid nanocapsules (GemC_12_-LNC) hydrogel is injectable, well tolerated, and has adequate properties for an application in the brain [11]. Following administration in the post-surgical cavity, it is able to delay tumor growth in several rodent models [12, 13], potentially filling the therapeutic gap between surgery and standard-of-care chemoradiation.

However, administering GemC_12_-LNC does not prevent long-term GBM relapse. A better understanding of the physiological and cellular factors that contribute to tumor regrowth would therefore be a prerequisite for developing accurate combinatory regimens that target residual tumor cells and the post-surgical microenvironment (SMe). Indeed, the brain response following GBM surgery is characterized not only by physiological regenerative responses that are beneficial for the healing process but also by distinctive time-dependent peritumoral immune responses exerting glioma-supportive effects [4, 17, 18]. Environmental changes such as blood–brain barrier (BBB) disruption, glutamate imbalance, neuron death and failure to resolve inflammation modify the state and interactions between populations that co-exist at surgical margins [4, 19–22]. Alieva *et* al. reported that less invasive procedures, such as needle biopsies, can increase tumor volume in GBM patients ultimately resulting in negative effects of traumatic lesions on tumor growth [23]. However, the clinical data on the impact of more invasive surgical procedures on recurrences are limited, as the post-surgical follow-up of patients aims at selecting the most appropriate therapeutic plans rather than exploring the local microenvironmental changes following surgery.

The tumor microenvironment is composed of tumor cells and non-tumor cells (*e.g.,* immune, vascular and resident brain cells) whose crosstalk contributes to tumorigenesis, tumor progression and treatment resistance. The GBM tumor microenvironment is a promising therapeutic target, and recent methodological advancements have improved our understanding of the microenvironment of primary and recurrent tumors following radiotherapy and chemotherapy [24, 25]. Tumor-associated macrophages/microglia (TAMs) are the most abundant immune cells in GBM. TAMs gather brain resident immune cells, microglia and border-associated macrophages (BAMs), and peripherical myeloid cells including monocytes, and monocyte-derived cells [26–28] and several strategies have been tested in the recent years to target them in primary tumors [28, 29]. Few studies have characterized the immune responses following GBM surgery in animal models, mainly in the context of the development of immunotherapies [17, 30–33]. For example, we demonstrated that acute post-surgical inflammation could enhance vaccine-induced immune responses to reduce recurrences, highlighting the SMe as a therapeutic target [32]. Recently, it was shown that partial tumor resection induces a fibrotic scar adjacent to the surgical margins which can promote tumor cell survival. This fibrotic response was also linked to the resistance to anti-colony-stimulating factor-1 receptor immunotherapy and radiotherapy [34]. However, an accurate and dynamic characterization of the cell composition, phenotypes and activation states of the SMe is needed to reveal time frames and key mediators that can be exploited and targeted for a therapeutic effect.

In this work, we used a clinically relevant “biopsy punch” surgical technique that we previously developed to resect orthotopic GBM tumors in rodents [15, 32, 35], to longitudinally characterize the SMe from surgery to recurrence. Single-Photon Emission Computed Tomography (SPECT) imaging was performed post-surgery to evaluate BBB permeability via [99mTc]Tc-DTPA SPECT/CT [36]. Transient BBB disruption post-surgery was also confirmed by magnetic resonance imaging (MRI) in GBM patients. Multiparametric flow cytometry was used for immunophenotypic analysis of blood cells to evaluate the systemic immune landscape following surgery in mice. Biphotonic imaging and spatial hyperplexed immunofluorescence imaging were used to unravel the complexity of the SMe at identified time frames, and the morphology, spatial organization and interaction of the immune cells around the resection cavity. 3D brain analysis confirmed that unresected tumors and post-surgical recurrences differ in terms of density, morphology and interactions between the immune cells composing their tumor microenvironment. Moreover, immunophenotyping of the tumors allowed the identification of cellular targets that are overexpressed in post-surgical recurrences compared with unresected tumors. On the basis of these observations, we propose a therapeutic strategy combining local treatment with the GemC_12_-LNC chemotherapy-based hydrogel and systemic administration of an immunomodulatory drug to delay the onset of recurrence. Our preclinical results hold promise for the clinical translation of this multifaced treatment as it efficiently fills the currently unused therapeutic window between surgery and chemoradiation.

## Materials and methods

### Retrospective study on patients

The perioperative cohort was a local prospective cohort composed of 10 adults (≥ 18 years) *IDHwt* GBM patients followed at La Timone Hospital (Assistance Publique — Hôpitaux de Marseille, AP-HM, France). For these patients, a brain MRI (with at least T1, T1 with gadolinium, T2 and FLAIR sequences) was performed at the time of diagnosis, within 48 h after surgical resection and every two months until progression.

The following data were recorded: age, sex, Karnofsky performance status (KPS), histology, type of surgery, oncological treatment, MRI characteristics and topography of relapse.

All patient data were obtained according to a protocol approved by the local institutional review board and ethical committee (PADS 20–343). All the patients supplied written informed consent. The present study was conducted in accordance with the Declaration of Helsinki.

### Characterization of the post-surgical microenvironment in mouse models

The in vivo experiments reported in this work for the GL261 model were approved by the institution’s Animal Care and Use Committee (CE71, Aix-Marseille Université, reference n° 22185) and performed following the French national regulation guidelines in accordance with EU Directive 2010/63/EU. The mice were housed in enriched cages in a temperature- and hygrometry-controlled room, had free access to water and food and were monitored daily. The mice were immunocompetent seven-week-old female C57BL/6JOlaHsd mice (for nuclear imaging, immunophenotyping, brain clearing and efficacy studies; Envigo, Gannat, France) or double heterozygote transgenic LysM-EGFP//CD11c-EYFP reporter mice (for biphotonic imaging and immunohistochemistry) [26]. The animals were sacrificed at the end of the study (*e.g.* 14 days- post-surgery, 130 days post-grafting for long-term survivors) or when they reached the end-points (≥ 20% body weight loss or 10% body weight loss plus clinical signs of distress e.g., paralysis, arched back, or lack of movement for glioma-bearing animals).

#### Orthotopic glioma mouse model

For tumor grafting, the animals were anesthetized with ketamine/xylazine (100 and 10 mg/kg intraperitoneal injection, respectively) and fixed in a stereotactic frame. The skin surface on the head was disinfected with antiseptic solution (Vétédine^®^ solution, Vetoquinol, Lure, France) and the hair was removed. Lidocaine (10 mg/mL; Aguettant, Lyon, France) was injected subcutaneously at the site of the incision, and the eyes were protected with an ophthalmic gel (Ocry-gel, TVM lab, Lempdes, France). To hydrate the animals, 1000 μL of physiological solution (0.9% sodium chloride; Aguettant, France) was injected subcutaneously into the flank. An incision was made along the midline, and a burr hole was drilled into the skull at the parietal lobe (2 mm posterior and lateral to the bregma) *via *a high-speed drill (Tack Life Tools, New York, USA; 0.8 mm diameter round end engraving burrs: Dremel, Breda, The Netherlands). A 10 μL 26 s gauge syringe with a cemented 51 mm needle (Hamilton, Rungis, France) was used to inject 1 × 10^5^ GL261-DsRed glioma cells—cultured as previously reported [26]—into the cortex (1 mm deep from the outer border of the brain) via an automatic pump device at a speed of 0.2 μL/min. The wound was then closed* via* tissue adhesive glue (3M Vetbond^®^, Sergy-Pontoise, France), and the animals were allowed to recover under an infrared heating lamp.

#### Tumor resection and cranial window implantation

Fourteen days post-grafting, the animals were anesthetized with ketamine/xylazine (100 and 10 mg/kg intraperitoneal injection, respectively), fixed again in a stereotactic frame and prepared for surgery as previously described. With scissors, the skin along the midline of the previous surgical scar was cut to expose the skull. The periosteum was removed with fine tweezers to reveal the bregma and ensure adherence of the cranial window. A 5 × 5 mm circular cranial window was then defined in the left parietal bone around the previous injection hole to expose the brain. The skull was gently lifted and removed passing the tweezers between the brain and skull to avoid removal of the tumor attached to the skull. The presence of the tumor was confirmed *via* fluorescence microscopy.

To resect the tumor, the biopsy punch technique previously developed and validated by the authors was used [35]. Briefly, a biopsy punch (2 mm Ø, Kai Medical, Germany) was placed around the injection hole and inserted slowly 2 mm deep, followed by gentle twisting to cut the brain/tumor tissue. A Pasteur pipette connected to a vacuum pump was used to remove the explant and build up blood. Hemostasis was achieved by inserting an absorbable hemostatic triangle (Sugi^®^ Sponge Points Kettenbach, Questalpha GmbH, Germany) into the formed cavity. The dural cranial window was covered with a round glass coverslip (5 mm Ø, 0.15 ± 0.02 mm thickness; Warner Instruments, USA) glued with histocompatible acrylic glue (Cyanolit, AC Marca, France). The window was then sealed on the adjacent bone and fixed to the skull with dental cement (Unifast Trad™, GC America, USA). The control animals either received cranial window implantation at the day of cells grafting (cranial window control) or on day 14 post-grafting (unresected control) but did not receive biopsy punch tumor debulking. The presence or absence of tumors post-cranial window implantation and/or post-surgery was observed *via* fluorescence microscopy.

Survival analysis and immunohistochemistry were also performed in a patient-derived GBM9 [37] xenograft orthotopic resection model without cranial window implantation. Please refer to supplementary data for the methodology used for this animal model.

#### Blood immunophenotyping by flow cytometry

To evaluate the systemic immune response following surgery, complex cellular phenotyping was performed on blood cells the day before surgery and cranial window implantation and 3, 7 and 10 days later. The animals were anesthetized with 1.5% vol% isoflurane (IsoVet^®^, Laboratoire Osalia, Paris, France) and approximately 120 µl of blood was collected by retro-orbital sampling via K2 EDTA precoated capillaries for microhematocrit (Vitrex^®^, Vitrex Medical, VWR, France) and stored at room temperature in Eppendorf tubes.

For erythrocyte lysis, lysis solution (eBioscience™ 1X RBC Lysis Buffer, Invitrogen, USA) was added to each blood sample, mixed and stored at room temperature for 5 min. Then, the reaction was stopped by adding a buffer solution consisting of Dulbecco’s Phosphate buffered saline (DPBS 1X no calcium no magnesium, Gibco^TM^, Thermo Fisher Scientific, France) with 0.5% Bovine Serum Albumin (BSA Fraction V, Eurobio Scientific, France) and 2 mM EDTA (Invitrogen, Life Technologies, USA) in each tube, which was subsequently centrifuged (1200 rpm, 4°C for 10 min). For antibody staining, the supernatant was removed and an appropriate volume of BD Horizon™ Brilliant Stain Buffer (BD Biosciences, France) was added to the pellet. Appropriate dilutions of the antibodies (Table S1) were prepared in buffer solution and the antibody mixture was added to the cell suspension and incubated in the dark at 4°C for 20 min. Then, the cells were washed, pelleted by centrifugation and appropriately suspended in buffer solution for flow cytometry acquisition (Cytoflex LX instrument, Beckman Coulter, France) at the AMUTICYT core facility of Aix-Marseille Université. Data were acquired using Cytexpert 2.2 software. Dimensionality reduction was performed using opt-SNE in OMIQ after data cleanup on manually gated viable singlet leukocytes CD45 + . The 10 channels were selected for running the algorithm: CD45, Ly6G, CD161, Ly6C, CD357, MHCII, CD11b, F Siglec, CD8 and CD11c. The advance settings were set to the default mode of the OMIQ software (Max iterations 1000, Perplexity 30). Dimensional reduction was completed after 560 iterations. The Opt-SNE representation shows the manually selected cell population using the gating strategy illustrated in Figure S3A. Thus, to validate the manual selection of immune cell clusters, the FlowSOM algorithm using Elbow metaclustering was performed on the 10 channels and optSNE_1 and optSNE_2.

#### Biphotonic imaging studies

Image acquisition was conducted, as previously described [26], at days 7 and 14 post-surgery and cranial window establishment. Prior to each imaging session, the mice were anesthetized with ketamine/xylazine (100 and 10 mg/kg respectively, *via* intraperitoneal injection) and injected intravenously with 100 μL of a quantum dots solution (QDots; Qtracker™ 705 Vascular Labels, ThermoFisher, 6 μg/100 μL in DPBS) and positioned on a stereotaxic frame allowing movements in the three-directions.

For the acquisitions, a Zeiss LSM 780-MP 2-photon microscope home modified to allow animal positioning below the 20 × water immersion objective (1.0 NA) and coupled to a femtosecond pulsed infrared tunable laser (Chameleon Ultra 2, Coherent) was used. Images were acquired using an excitation wavelength tuned at 920 nm to simultaneously excite all the fluorophores. The signals were epicollected and separated by dichroic mirrors and filters on five independent non-descanned detectors. Gains and offsets were identical for all the detectors, except for the red channels whose gain was reduced by 30% to compensate for the strong expression of DsRed in tumor cells. Images were acquired below the dura matter over a depth of 500 μm using 10 μm steps. The laser power was linearly increased with depth. Z-stack images were acquired as mosaics (stitching mode) to cover the whole tumor surface.

For the biphoton image analysis, only horizontal plans were considered. Spectral unmixing was first applied to raw 2-photon images (Zen software) and we exploited the different Imaris microscopy image analysis software v10.1 (Bitplane, Oxford Instruments) options to perform all the further analyses. The corresponding images at day 7 and day 14 post-surgery were aligned according to the vasculature structures. The volume of each tumor was defined by creating a 3D mask *via* the surface tool. Cells found in blood vessels were easily excluded thanks to a 3D mask associated to the vasculature, and then reconstituted with a 3D mask for each cell population. Hence, to assess the different cell densities, the reported numbers of fluorescent positive cells (LysM-EGFP^+^, CD11c-EYFP^+^ and LysM-EGFP^+^/CD11c-EYFP^+^) were reported over the total volume. Colocalization analyses were performed to identify LysM-EGFP^+^/CD11c-EYFP^+^ double-labeled cells. The morphology and area of individual cells were analyzed with the sphericity score. To measure the distance of LysM^+^ cells from CD11c^+^ cells, the shortest distance from each cell was analyzed.

#### Nuclear imaging studies

##### Single Photon Emission Computed Tomography (SPECT) Imaging

BBB opening was evaluated by SPECT/CT imaging using [99mTc]Tc-DTPA as a radiotracer on day 13 post-grafting (day prior to surgery, controls) and at day 1, 2, 3, 7, and 10 post-surgery. The controls were all tumor bearing animals without or with cranial window implantation on the day of cell grafting.

[99mTc]Tc-DTPA was administered* via *intravenous injection of 50 μL of radioactive tracer (30 MBq) in an isotonic and pyrogen-free solution using an insulin syringe (27G) [36]. Thirty minutes after injection, SPECT/CT acquisition was performed for 20 min under anesthesia with 1.5% vol% isoflurane (IsoVet^®^, Laboratoire Osalia, Paris, France) using a NanoSPECT/CTplus^®^ camera and the Nucline^®^ 1.02 acquisition software (Mediso Medical Imaging System Ltd., Budapest, Hungary). SPECT and CT DICOM files were fused for reconstruction and image processing was carried out with VivoQuant^®^ 3.5 and InvivoScope^®^ 2.00 reconstruction software (InviCRO, Boston, MA, USA) to assess tracer uptake in the brain.

### Characterization of the microenvironment of primary *vs* recurrent tumors

#### Histological analysis

Immunofluorescence was performed on brain slices obtained from transgenic mice used for biphotonic imaging. Ly6G staining was performed to identify neutrophils among LysM-EGFP^+^ cells, and TMEM119 staining was performed to discriminate between dendritic cells and basal microglia among CD11c-YFP^+^ cells. Animals were deeply anesthetized at the appearance of clinical symptoms by xylazine/ketamine (100 and 10 mg/kg intraperitoneal injection, respectively). Then, they were perfused by cardiac injection of 10 mL of DPBS 1X and then 10 mL of paraformaldehyde (PFA 4% aqueous solution, methanol free; Electron Microscopy Sciences, UK). The brains were extracted and embedded in OCT embedding matrix (CellPath, Newtown Powys, United Kingdom), and 8 µm thick sections were prepared using a cryostat CM 3050S (Leica). The slices were then dried at 55°C for one hour, and after being returned to room temperature, they were stored at -20°C until use. For immunofluorescence, the slices were incubated in methanol for 1 h and then rinsed (for TMEM slices), and/or a 2 h blockage of non-specific sites was performed in saturation medium (BSA 5%, goat serum 5%, donkey serum 5%, Triton 0.3%). Primary antibodies were then incubated for 1 h (Anti-TMEM119: Abcam, 1/100, ab209064) or overnight (Anti-Ly6G: Biolegend, 1/100, 127610, coupled with A647) at room temperature in saturation medium. For the TMEM slices, secondary antibody (anti-rabbit Alexa Fluor 647; Biolegend, 1/1000; 406414) was then incubated 1 h at room temperature with Hoechst (Sigma-Aldrich, 1/1000). The Ly6G slices were incubated with Hoechst for 15 min. The slices were then incubated with copper sulfate/ammonium chloride for 10 min and then mounted with Prolong Mounting Medium (Sigma-Aldrich, France). Images were acquired with a Zeiss LSM700 or LSM780 confocal microscope.

Hematoxylin eosin staining and immunohistochemistry were performed on brains derived from the GBM9 xenograft orthotopic resection model. After sacrifice, brains were extracted and fixed in 10% formalin solution (Merck, Germany) for 24 h and then stored in PBS at 4 °C. Brains of at least three mice per group were embedded in paraffin, sectioned in 10 μm sections using a RM2255 microtome (Leica, France) and collected on super-frost plus glass slides. Slides were incubated at 37 °C overnight and then stored at room temperature until further use. The presence and location of the tumors as well as cellular inflammatory responses was confirmed in the brains by histological analysis. For this, samples were deparaffinized and stained with anti-Ki67 (Roche, Switzerland) on a Benchmark Ventana autostainer (Ventana Medical Systems SA, Illkirch, France) or manually with Hematoxylin and eosin (H&E), anti-Iba-1 (Wako, 1/500, Ao19-19,741), and anti-Arginase1 (Aviva Systems Biology, 1/50, ARP45673) according to manufacturer’s instructions and as previously described [38]. Slides were then scanned (Nanozoomer 2.0-HT, Hamamatsu Photonics SARL France, Massy, France) and images processed in NDP.view2 software (Hamamatsu). Results of the staining were determined based on the areas with tumor cells (*n* = 3).

#### 3D analysis of the brain and tumor microenvironment

For 3D analysis of the brain and tumor microenvironment, animals were deeply anesthetized at 14 days post-surgery and/or cranial window implantation with xylazine/ketamine (100 and 10 mg/kg intraperitoneal injection, respectively) and the presence of the tumors was evaluated *via* fluorescence microscopy. Then, they were perfused by cardiac injection of 10 mL of 1X DPBS and then 10 mL of PFA. The brains were extracted and placed in PFA for 24 h at 4 °C, and then stored in PBS containing 0.02% sodium azide (Sigma Aldrich) at 4 °C until further use.

Hemi brains were processed following the iDISCO^+^ protocol as previously described [39] and routinely performed in our laboratory [40]. Briefly, the brains were dehydrated in successive methanol baths, placed in a solution of 66% dichloromethane (Sigma Aldrich) and bleached with 5% H_2_O_2_ (Sigma Aldrich). The samples were subsequently rehydrated in sequential baths of methanol and permeabilized (PBS/0.2% TritonX-100 (Euromedex)/20% DMSO (Sigma Aldrich)/0.3 M glycine (Euromedex)) at 37°C for 2.5 days. The samples were then incubated in blocking solution (PBS/0.2% TritonX-100/10% DMSO/3% donkey serum (Jackson Immunoresearch)) at 37°C for 4.5 days and incubated for 5 days in primary antibody solutions (Table S2; PBS-Tween 0.2% with 10 mg/mL heparin (Sigma Aldrich)/5% DMSO/3% donkey serum) at 37°C for 5 days. After washing, the samples were incubated with secondary antibodies (Table S2) in PBS-Tween 0.2% with 10 mg/mL heparin /3% donkey serum at 37°C for 5 days. The samples were finally dehydrated in successive baths of methanol and cleared in a BABB solution (33% Benzyl alcohol, Sigma Aldrich, France; 66% Benzyl benzoate, Fischer Scientific, France) for 24 h and then stored in BABB until light sheet acquisition.

The samples were imaged in a MACS imaging solution (Miltenyi Biotec, Germany) in a sagittal orientation with an Ultramicroscope BlazeTM (Miltenyi Biotec) equipped with a 4.2 Megapixel sCMOS camera and 1.1 × /4 × /12 × objectives. A numerical aperture of 0.1/0.35/0.53 was used with fixed 4 source illumination. The microscope was equipped with LED lasers (488 nm, 561 nm and 639 nm). The emission filters used were 525/50, 595/40, and 680/30. The samples were scanned with sheet of 5 µm of thickness (for the 1 × and 4 × objectives). A horizontal dynamic correction was applied when mosaics were performed with the 4 × objective.

Images were analyzed as 3D projections *via* Imaris software as previously described [40]. The volume of each tumor was defined by creating a 3D mask using the surface tool. Only cells present in the tumor mask were segmented according to the local intensity contrast. For brains analysis, to assess cell density, the number of TMEM119^+^ cells were reported to the tumor volume. To measure the distance from the tumor center and vessels, each cell was dotted with the spot tool according to the local intensity contrast, and the shortest distance from tumor center or vessels, reconstructed in 3D with the surface tool, was analyzed.

#### Tumor immunophenotyping by flow cytometry

To evaluate the local immune landscape of recurrent tumors, complex cellular phenotyping was performed on tumors extracted at day 10–14 post-surgery and/or cranial window implantation in wild-type mice bearing tumors. The animals were deeply anesthetized with xylazine/ketamine (100 and 10 mg/kg *via* intraperitoneal injection, respectively) and perfused *via* a cardiac injection of 20 mL of 1X PBS. The brains were extracted, and the cerebral hemisphere containing the tumor was excised using fine tweezers. The tumor and surrounding healthy cerebral cortex were dissociated in gentleMACS™ tubes (Miltenyi Biotec) on a GentleMACS Octo Dissociator (Miltenyi Biotec) using a Brain Tumor Dissociation Kit (Miltenyi Biotec). The suspension was then filtered through 70 µm MACS^®^ Smart Strainers (Miltenyi Biotec). For erythrocyte lysis, lysis solution was added to each sample and stored at room temperature for 3 min. Then, the reaction was stopped by dilution in buffer solution (DPBS with 0.5% BSA and 2 mM EDTA). The cell pellet was then suspended in buffer solution containing FcR blocking reagent mouse (Miltenyi Biotec) and anti-CD45 microbead mouse (Miltenyi Biotec) and incubated for 15 min at 4 °C. For magnetic cell sorting, LS columns (Miltenyi Biotec, Germany) were used to separate CD45^+^ cells from the total suspension. For antibody staining, an appropriate volume of BD Horizon™ Brilliant Stain Buffer (BD Biosciences, France) was added to the pellet of CD45^+^ cells. Appropriate dilutions of the antibodies (Table S3) were prepared in buffer solution and the antibody mixture was added to the cell suspension and incubated in the dark at 4 °C for 20 min. The cells were subsequently rinsed, pelleted and appropriately suspended in buffer solution for flow cytometry acquisition with a Cytoflex LX instrument. Data were extracted *via* Cytexpert 2.2 software and analyzed using Kaluza analysis software. The gating strategy used to extract and separate immune cells is illustrated in Figure S8-9.

#### Spatial hyperplexed immunofluorescence imaging

For multiplexed spatial imaging, animals were sacrificed at day 3, 7 and 10 post-surgery and brains were snap-frozen in isopentane (Sigma, France) precooled in liquid nitrogen and stored at -80°C until further use. Brains were then embedded in OCT (Cell Path, Newton Powys, United Kingdom) and sectioned with a thickness of 12 µm using a cryostat (Leica, France), carefully placed on a Superfrost Plus microscope slide (Thermo Scientific, France), and stored at -20°C. The day of the experiment, slides were processed for fixation with 4% PFA (Electron Microscopy Sciences, Hatfield, Pennsylvania, USA) and stored at 4°C in MACSima System Buffer (Miltenyi Biotec). Subsequently, slides were mounted in a MACSwell Four Imaging Frame (Miltenyi Biotec), and samples were stained with DAPI for 10 min. Slides were then placed in the MACSima Imaging System to start the MICS run. Antibodies used are listed in Supplementary Table 4. Antibody working solutions were prepared in MACSwell Deepwell Plates (Miltenyi Biotec) and sealed with MACSwell Sealing Foil (Miltenyi Biotec). One region of interest (ROI) per tumor was selected. Raw data were processed for registration and background correction. The pre-processed dataset was then analyzed in MACS iQ View Analysis Software (Miltenyi Biotec).

### Exploiting the post-surgical microenvironment as a therapeutic target

#### In vitro cellular studies

To propose an innovative therapeutic combination that targets both residual tumor cells and the SMe, we first analyzed the effects of the selected drugs in combination on GL261, GBM9 and CT2A cells to exclude any antagonistic effects. GL261 (DSMZ, Germany) and CT2A (Millipore, USA) mouse glioblastoma cell lines were cultured in DMEM (high glucose, pyruvate; Gibco™, Themo Fischer Scientific, France) supplemented with 10% FBS (Eurobio Scientific, France) 1% 10,000 U/mL penicillin G and 10,000 μg/mL streptomycin (Sigma-Aldrich, France). GBM9 human-derived glioma stem cells [37] were cultured in serum-free stem-cell permissive medium composed of DMEM/F-12-Ham medium in a 3:1 ratio supplemented with hormones (0.5 μg/ml insulin, 20 nM progesterone, 100 μM putrescine, 100 μg/ml transferrin), growth factors (10 ng/mL FGF; 20 ng/mL EGF; 2% of B-27 supplement), 0.02% of 100 μM sodium selenite, 1% of 10000 U/mL Penicillin G and 10,000 μg/mL Streptomycin, 1% of 100 mM sodium pyruvate [41]. Cells were subcultured in 75 cm^2^ culture flasks as adherent monolayers at 37° C and 5% CO_2_, while GBM9 cells were maintained in culture in 25 cm^2^ culture flasks as floating spheres.

For the cytotoxicity experiments, GL261 and CT2A cells (5000 cells/well) were seeded in 96-well plates while GBM9 (4000 cells/well) were seeded on poly-DL-ornithine (Sigma-Aldrich) coated 96-well plates (10 µg/mL) to grow as monolayers. Cells were allowed to grow for 24 h before they were treated with GemC_12_-LNC, GDC-0152 (MCE® MedChem express, USA) and their combinations at different ratios. N-dodecanoyl gemcitabine (GemC_12_, Atlanchim Pharma, France) was formulated as a nanomedicine hydrogel as previously reported [9]. The concentrations selected for the drugs were based on the half minimal inhibitory values (IC_50_) previously obtained for each drug in each cell line. For the combinations, the doses selected for GemC_12_-LNC were IC_50_, ½ IC_50_ and ¼ IC_50_, and for GDC-0152 they were 2 × IC_50_, IC_50_, ½ IC_50_ and ¼ IC_50._ Growth inhibition of GL261 and CT2A cells was measured after 72 h of treatment via a colorimetric MTT assay (Sigma-Aldrich) for GL261 and CT2A, or by Titer-Glo^®^ luminescent cell viability assay (Promega, France) for GBM9. The results obtained were normalized to those of the untreated controls and analyzed using the SynergyFinder web application to generate heat synergy maps and Highest Single Agent (HSA) synergy scores [42, 43]. The experiments were performed in triplicate.

#### Tumoroids culture and analysis by flow cytometry

Fresh GBM IDHwt tumor specimens were collected at AP-HM after surgery and placed in Hank’s Balanced Salt Solution. Samples were obtained from the center of biological resources of AP-HM (CRB BB-0033–00097) according to a protocol approved by the local institutional review board and ethics committee (2014-A00585–42) and conducted according to national regulations (NCT06045065). The study was performed in accordance with the declaration of Helsinki. All the patients provided written informed consent. All samples were processed within the 4–24 h following surgery. Tumoroids were cultured as previously described [44].

Tumoroids were treated with DMSO, GDC-0152 (100 µM), GemC_12_-LNC (25 µM) or a combination of both. After 72 h of treatment, tumoroids were dissociated using accutase (Thermo Fisher Scientific) and stained for flow cytometry. The antibodies used for the phenotyping are listed in Supplementary Table 5 according to the manufacturer’s instructions (Miltenyi Biotec). Doublets were removed and living cells were isolated using Viability 405/452 (Miltenyi Biotec). Cells were processed on MACSQuant^®^10 flow cytometer (Miltenyi Biotec) and data were analyzed using Kaluza (Beckman Coulter).

#### Anti-tumor efficacy studies

To evaluate the anti-cancer efficacy of our proposed combination regimens under clinically relevant conditions, we used a preclinical model previously developed and validated in our laboratory to resect GBM tumors [35]. In this model, animals were grafted with 1 × 10^5^ GL261-DsRed cells as described above but the injection coordinates were 2 mm posterior and lateral from bregma, 2.2 mm deep from the outer border of the brain. On day 14 post-tumor inoculation, all the mice underwent tumor resection and were randomly assigned to one of the following groups: 1) untreated (*n* = 9); 2) GDC-0152 (*n* = 9); 3) GemC_12_-LNC (*n* = 8); or 4) GemC_12_-LNC and GDC-0152 (*n* = 8). Tumor surgery was performed as previously described but this time the biopsy punch was inserted at a depth of 3 mm to allow the local injection of therapeutic concentrations of hydrogel at the time of surgery [12, 13]. For groups 1 and 2, no treatment was administered in the tumor cavity. For groups 3 and 4, the GemC_12_-LNC hydrogel was injected into the resection cavity *via* a 0.3 mL insulin syringe (GemC_12_ dose: 4 mg/kg, 5 μl of GemC_12_-LNC hydrogel). For groups 2 and 4, the monovalent SMAC mimetic GDC-0152 (Selleckchem, USA) was dissolved in DMSO (Sigma-Aldrich, France) and administered by retro-orbital injection at a dose of 20 mg/kg (100 µl in NaCl 0.9%) [38, 40]. The GDC-0152 treatment was performed post-surgery and every week (8 administrations in total). Following surgery and local treatment administration, the dural window was repaired by covering it with a 4 × 4 mm square piece of Neuro-Patch^®^ (Aesculap, Germany) impregnated with fibrin sealant (Tisseel Prima; Baxter, France). The wound was then closed with tissue adhesive glue. The mice were then monitored daily, and their body weights were measured 2–3 times per week. The mice were sacrificed when they reached the endpoints.

#### Immunohistochemistry

The brains of animals subjected to different treatment conditions and sacrificed within the same time window (between day 25 and 47 post-grafting, *n* = 4 per group) were perfused and stored in PBS as previously described until further use. For immunohistochemistry analysis, the brains were then embedded in 4% agar (Sigma-Aldrich, France) and 50 μm slices were obtained using a vibratome (Leica VT1200 S). The slices were immunostained against TMEM119 (for microglia), MHCII (for antigen-presenting cells, APC), Arginase 1 (ARG-1, for anti-inflammatory cells).

For double TMEM119/MHCII labeling, brain sections were incubated for 2 h at room temperature in blocking buffer (5% donkey serum, 5% BSA and 0.03% Triton 100X in 1X PBS). Sections from 4 different mice per condition were incubated with monoclonal anti-TMEM119 primary antibody (Abcam, 1/100; ab209064, clone 28–3) overnight at 4°C. The sections were then rinsed with 5% PBS/BSA (3 × 10 min), after which they were incubated with a monoclonal anti-MHCII primary antibody (Ebiosciences, 1/50; 14–5321-85) for 1 h at room temperature. For ARG-1 labeling, brain sections were permeabilized with denaturation buffer (10% PBS 10X, 16% HCl 37%, 0.5% Triton 100X in distilled H_2_O) at 37°C for 20 min and then incubated with neutralization buffer (3.8% sodium tetraborate in distilled H2O, pH adjusted to 8.5) for 10 min at room temperature. The slices were then rinsed with 5% PBS/BSA (3 × 10 min at room temperature) and incubated in blocking buffer. Anti-Arginase 1 polyclonal primary antibody (Novus Bio, 1/100; NB100-59740) was incubated at 4°C overnight. Following incubation with primary antibodies, the sections were washed with PBS/BSA 5% (3 × 10 min), incubated with anti-rabbit Alexa Fluor 488 (Invitrogen, 1/600; A11055), anti-goat Alexa Fluor 647 (Invitrogen, 1/600; A21447), anti-rat Alexa Fluor 647 (Jackson ImmunoResearch, 1/600; 712–605-150) and Hoechst (Sigma-Aldrich, 1/1000) secondary antibodies for 1 h at room temperature. After 3 × 10 min washes in 1X PBS, the sections were incubated in TrueBlack (Biotium, 1/20; 23,007) in 70% ethanol for 30 s. After a final wash (3 × 10 min), the slides were mounted with a mounting medium (Mowiol-488 /Glycerol/DABCO) and stored at 4°C. Confocal microscopy was carried out on a Zeiss LSM 700 microscope with a 20 × objective at 0.8 numerical aperture on 2 different tumor areas per slide (center and periphery). The images were then processed with ZEN Blue Edition version 3.0 and analyzed with Imaris software.

#### Statistical analysis

In the manuscript, “*n*” corresponds to the number of independent experiments performed (single animals for animal experiments), and the data are presented as the average ± SEM. Statistical analyses were performed using GraphPad Prism (GraphPad Software, USA). All reported *p*-values are two-sided and *p*-values < 0.05 were considered statistically significant (^#^*p* = 0.05, *p < 0.05, **p < 0.01, ***p < 0.001, ****p < 0.0001).

For the blood immunophenotyping analysis, multiple comparisons were performed using Kruskal–Wallis nonparametric analysis with uncorrected Dunn's test to compare the control and unresected/resected groups. Unpaired t test with Welch correction was used to compare unresected and resected groups at each timing. For brain immunophenotyping, data obtained from unresected and resected tumors were compared using a nonparametric Mann–Whitney test.

For the nuclear imaging quantification analysis, statistical analysis was performed using unpaired t test with Welch's correction at each time point *vs*. the control value.

For the biphotonic imaging quantification analysis, images obtained from at least three animals were analyzed using Imaris software and compared using paired nonparametric Wilcoxon test between the groups. To compare the quantification of the histological staining in unresected and recurrent tumors, the nonparametric Mann–Whitney test was used. The Chi2 test of independence was used to compare the repartition values of TMEM119^+^ cells within the tumors and their distance to blood vessels.

For the in vivo efficacy studies, the statistical analysis was estimated from a comparison of Kaplan–Meier survival curves using the log-rank test (Mantel-Cox test). To compare the quantification of the histological staining, unpaired t test was used.

## Results

### Evolution of the post-surgical systemic immune landscape in an orthotopic resection model

**Fig. 1:**
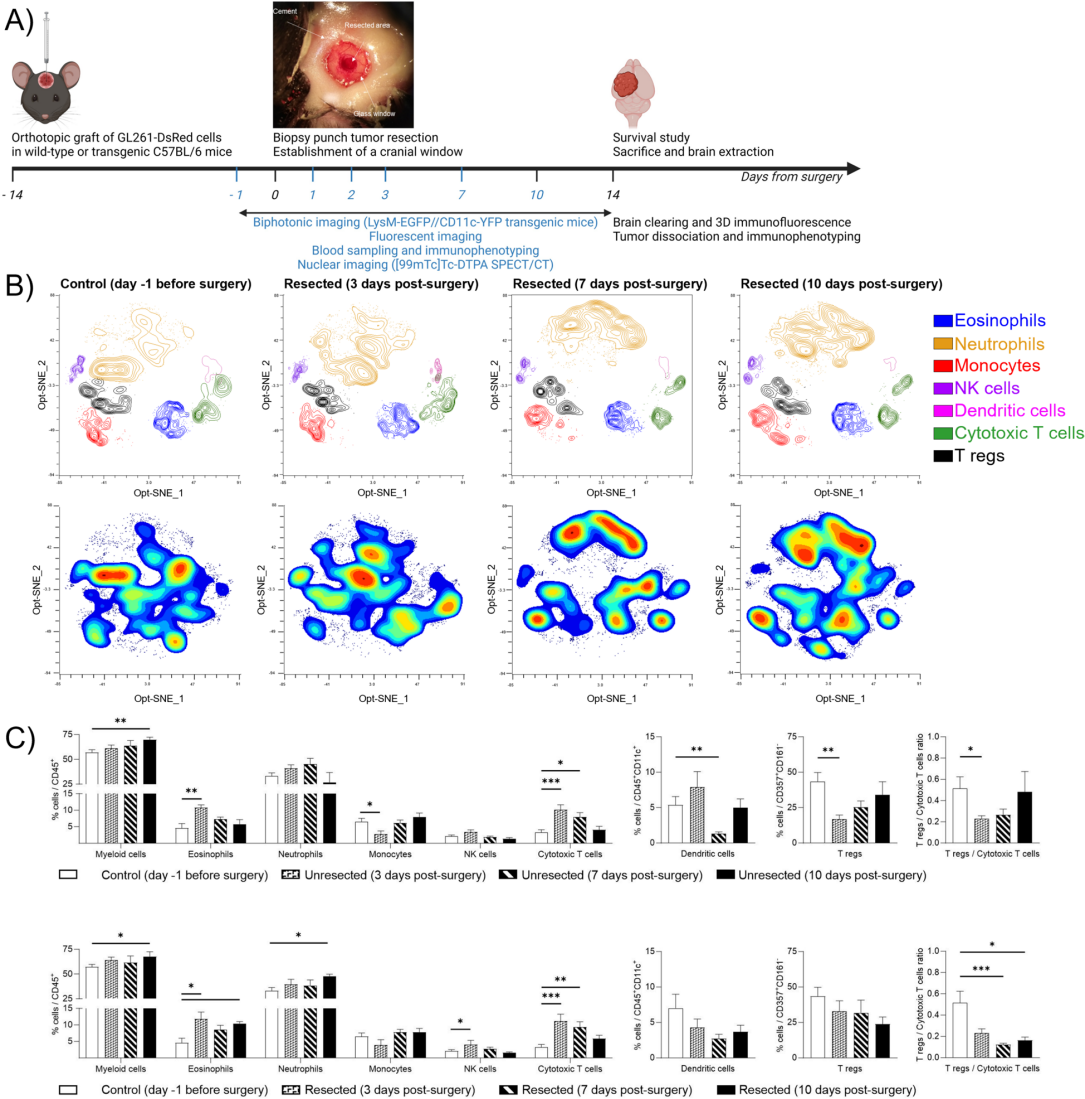
Longitudinal characterization of the impact of surgery on tumor recurrence: mouse model and systemic immune modifications. **A**) Time and work flow of the methods used in this paper to characterize longitudinally the systemic and local brain microenvironment from surgery to recurrence, as well as the post-mortem analysis performed to identify therapeutic targets in the microenvironment of recurrent tumors. The panel shows a light microscope image of the cranial window and resected area immediately after tumor resection; **B**) Identification of immune cell population in mice blood using flow cytometry. The upper panels show Opt-SNE of manually selected cell cluster validated by FlowSOM analysis for control and resected animals at day 3, 7 and 10 post-surgery. The lower panel represents, for the same groups of animals, the density contour plot of the different immune cell clusters in mice blood projected onto an Opt-SNE map; **C**) Frequency of repartition of blood immune cell clusters for control and unresected (upper panel) or resected (lower panel) animals at day 3, 7 and 10 post-surgery compared to day one before surgery. Numbers are expressed as percentage of cells related to CD45^+^ cells (for myeloid cells CD11b^+^, eosinophils, neutrophils, NK cells, Cytotoxic T cells), CD45^+^CD11c^+^ cells (for dendritic cells), CD357^+^CD161^−^ (for Tregs). The right panel represents the ratio between Tregs and Cytotoxic T cells. (average ± SEM, *n* = 5–9; Kruskal–Wallis nonparametric analysis with uncorrected Dunn's test, *p < 0.05; **p < 0.01; ***p < 0.001)

To monitor the systemic and local changes in the microenvironment over time from surgery to recurrence, we used a mouse resection model that we previously validated [13, 35]. To mimic GBM surgery, we grafted GL261-DsRed cells into either wild-type or transgenic C57BL/6 reporter mice [26] and performed a gross total resection using the “biopsy punch” technique [35]. A cranial window was applied to allow intra-tumoral optical imaging, while blood sampling and analyses of the tumoral tissue were performed over time or at the end of the experiment on the same animals (Fig. 1A). The animals did not show any signs of pain or distress following surgery and implantation of the cranial window. Recurrences were visible on day 7 post-surgery as individual tumor cells and formed a visible tumor mass within day 14 post-surgery (Figure S1A). Tumor debulking increased mouse survival by about 5 ± 0.5 days compared to animals which received cranial window without surgery (Figure S1B). Moreover, we observed that recurrences grew quickly reaching comparable volumes as the unresected tumors in a shorter time range (Figure S1C) suggesting the implementation of detrimental changes in the environment following debulking. To evaluate the impact of surgery on tumor cell proliferation, we used a xenograft human cancer-stem cell model, GBM9 [37, 41]. At the end point (7 months post-surgery), 37.5% of unresected animals and 50% of resected animals were still alive and without clinical symptoms (median survival 222.5 *vs* 271.5 days respectively, Figure S2A) but all brains presented a tumor (Figure S2B). Unresected and resected tumors were compared for cell morphology, proliferation, infiltration. Resected tumors presented more anisocariosis and undifferentiated cells. Unresected tumors were highly diffused without regions of high tumor cell density. Resected animals presented highly infiltrative tumor cells but also an identifiable dense tumor mass close to the resection cavity suggesting a different tumor development between unresected and resected tumors (Figure S2C).

To further explore the impact of surgery on the development of recurrences, we performed the dynamic investigations on the immunocompetent GL261 syngeneic model. Multiparametric flow cytometry analysis was performed on blood collected one day before surgery (craniotomy) and at days 3, 7 and 10 post-surgery (craniotomy with resection and/or cranial window implantation). For ethical reasons, we were unable to collect blood from the same animals before day 3 or at more than three time points. The panel that we constructed allowed the discrimination of different subpopulations of myeloid and lymphoid immune blood cells (Figure S3) which were then clustered *via* the unsupervised optimized t-Distributed Stochastic Neighbor Embedding (t-SNE) algorithm [45] (Fig. 1B and S4A, upper panels). In unresected and resected animals, a significant increase in the number of circulating CD11b^+^ myeloid cells—including eosinophils – and cytotoxic T cells was detected probably due to cranial window implantation, as no tumors were present in the resected group at the early times. We observed a return to basal levels within 10 days for all but CD11b^+^ myeloid cells (for both groups) and eosinophils (for resected animals only). The ratio of Tregs to cytotoxic T cells significantly decreased over time in both groups confirming the enrichment of cytotoxic T cells in the blood, but persisted at 10 days for the resected animals only. In unresected animals only, a significant increase in the number of dendritic cells and a decrease in the number of monocytes and T regs was quantified with return to basal values within 10 days. In comparison, a significant increase in circulating neutrophils and NK cells was quantified in resected animals with a return to basal value after 10 days only for NK cells. Overall, our results revealed a modulation of the systemic immune landscape in the first week following craniotomy due to implantation of a cranial window, which was ultimately modulated by tumor resection (Fig. 1C, S4B).

### Evolution of the local brain microenvironment from surgery to recurrence

**Fig. 2:**
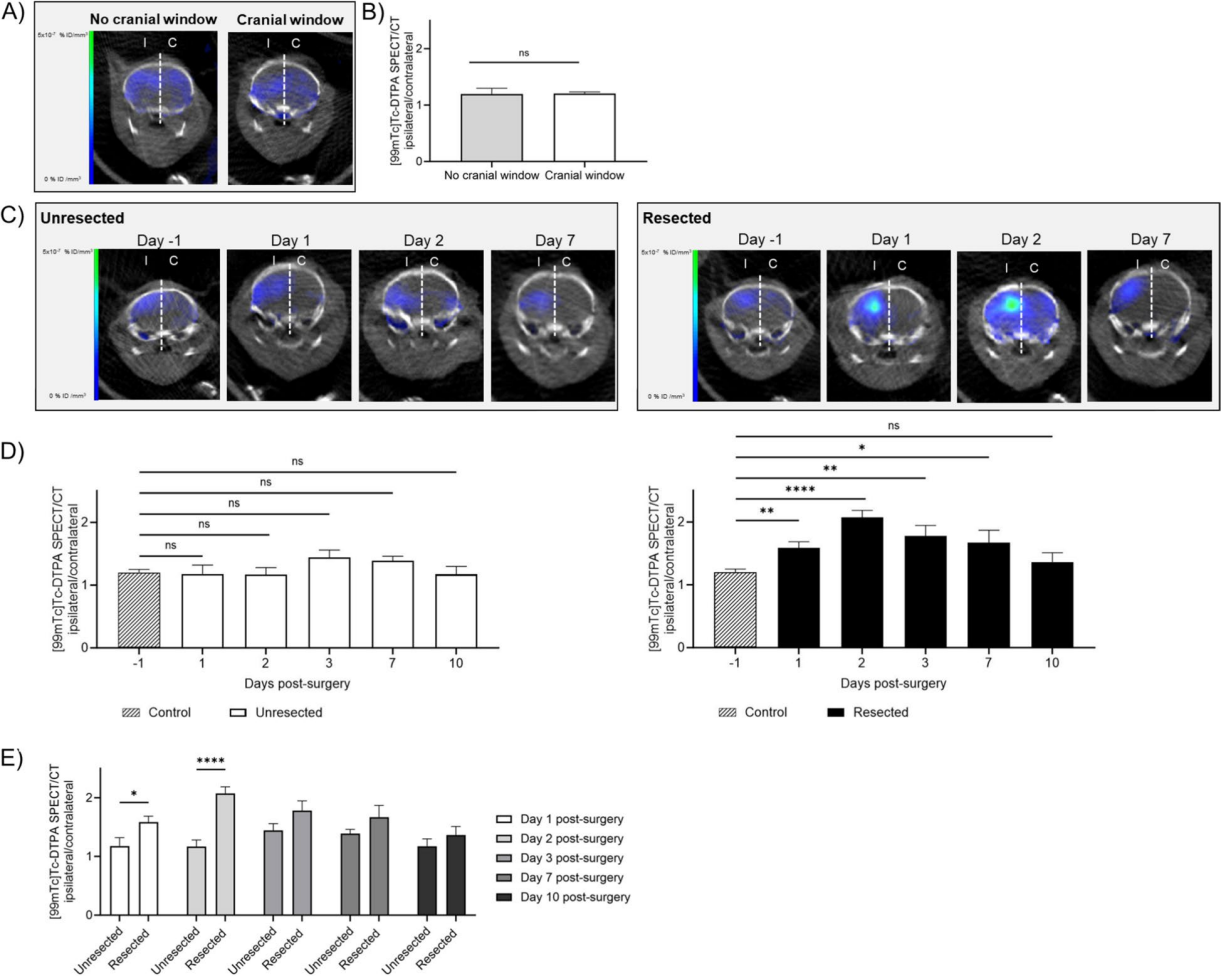
Longitudinal characterization of the impact of surgery on BBB permeability.** A**) Brain representative SPECT/CT tomographic images of [99mTc]Tc-DTPA distribution in animals that did not received cranial window implantation (no cranial window, left panel) or received cranial window implantation at the time of tumor cells grafting (cranial window, right panel) at day 13 post-grafting. The white dotted line represents the separation between ipsilateral (I) and contralateral (C) brain hemispheres that were used for the quantifications; **B**) Quantification of [99mTc]Tc-DTPA activity at day 13 post-grafting (one day prior to operation) in animals that did not received cranial window implantation (no cranial window) or received cranial window implantation at the time of tumor cells grafting (cranial window). These two groups of animals (representing the resection control and cranial window control, respectively) were then combined to establish the control group in panel D; **C**) Brain representative SPECT/CT tomographic images of [99mTc]Tc-DTPA distribution in animals that received cranial window implantation (unresected, left panel) or resective surgery and cranial window implantation (resected, right panel) 14 days following tumor cells grafting. Images were acquired at different times pre- and post-operation; **D**) Quantification of [99mTc]Tc-DTPA activity in control animals (imaging of control animals at day 13 post-grafting) *vs* animals 1, 2, 3, 7, 10 days post-implantation of the cranial window (unresected, left panel) or resective surgery and cranial window implantation (resected, right panel); **E**) Quantification of [99mTc]Tc-DTPA activity in animals 1, 2, 3, 7, 10 days post-surgery (cranial window implantation *vs* resection and cranial window implantation). For panels B, D and E panels, results are expressed as I/C ratio (*n* = 5–13, mean ± SEM; Unpaired t test with Welch’s correction; ns = not significant, *p < 0.05, **p < 0.01, ****p < 0.0001)

**Fig. 3:**
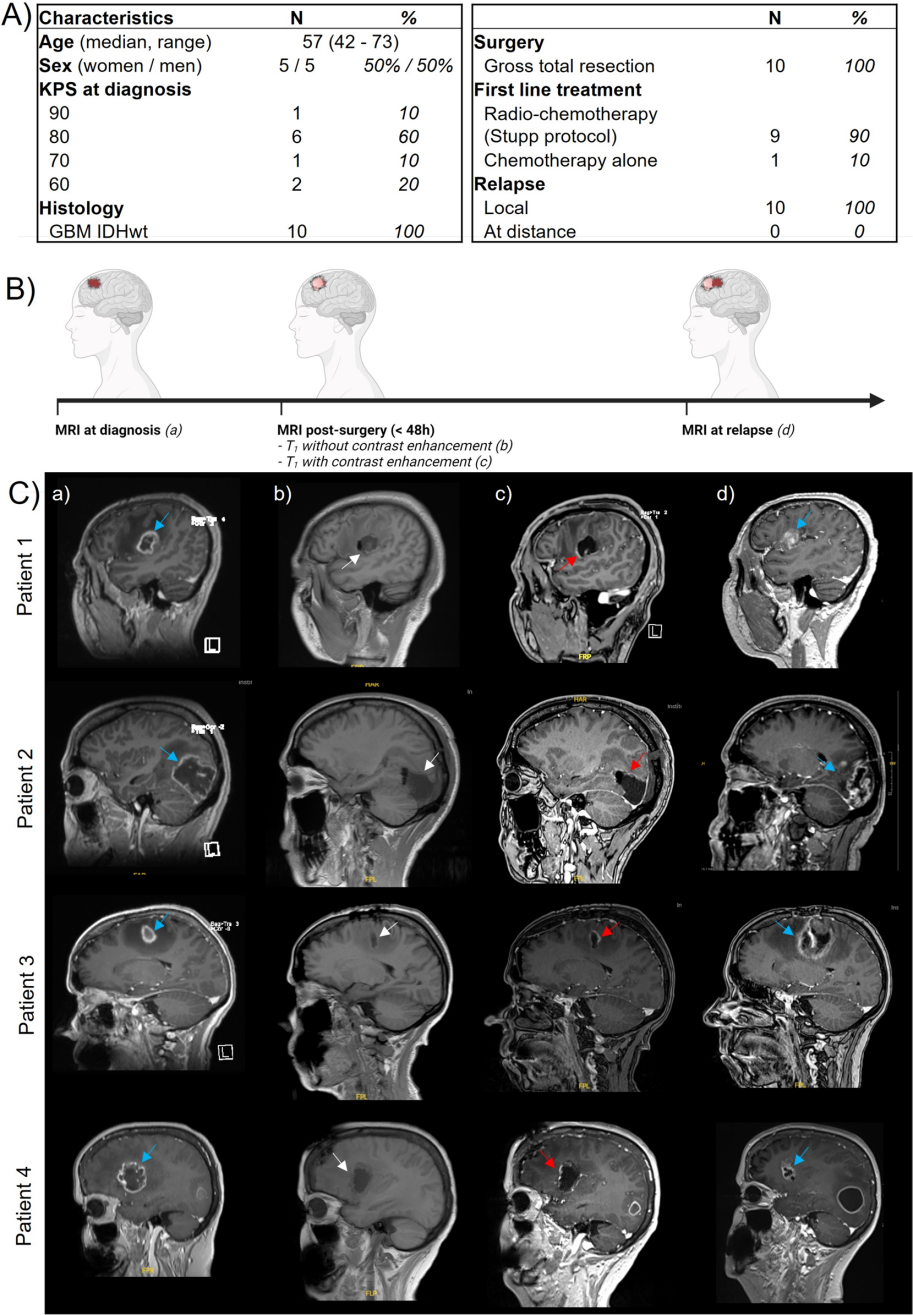
Surgery induces local modifications to the cerebral microenvironment in GBM patients. **A**) Patient characteristics of peri-operative cohort; **B**) Study design: GBM patients were enrolled in the study and magnetic resonance imaging (MRI) was performed at time of diagnosis, within 48 h post-surgery (without and with contrast enhancement) and at relapse; **C**) Illustrative brain MRI imaging of four patients showing a T1 sequence with (a, c, d) or without (b) contrast enhancement, before (a) and after gross total resection (b and c) and at relapse (d). The blue arrows show the initial and recurrent tumor location, the white arrows show the tumor cavity location (b) and the red arrows show contrast enhancement associated with post-surgery BBB opening (c)

To determine whether the modulation of the systemic response observed following cranial window surgery or tumor resection could have an impact within the brain, we evaluated BBB permeability by SPECT/CT imaging with [99mTc]Tc-DTPA. The presence of the cranial window did not induce BBB leakage (Fig. 2A-B). Moreover, the surgical implantation of the cranial window did not induce BBB permeability over time compared to animals at day -1 prior surgery (Fig. 2C-D, group unresected). On the contrary, quantification of [99mTc]Tc-DTPA showed a significant increase in ipsilateral/contralateral ratio signal at times 1, 2, 3 and 7 post-resection compared to control animals at day -1, with a return to basal signal at day 10 (-1 *vs* 1 *p* = 0.0462; -1 *vs* 2 *p* < 0.0001; -1 *vs* 3 *p* = 0.0048; -1 *vs* 7 *p* = 0.0162; -1 *vs* 10 *p* = 0.4725; Fig. 2C-D, group resected). The direct comparison between unresected and resected animals confirmed that the BBB permeability peaks at day 2 post-surgery (Fig. 3E). These results revealed a transient permeabilization of the BBB within the first week following surgery selectively on the hemisphere that received the tumor resection. These results highlight a key time window for immune cell brain recruitment and systemic drug administration.

### Surgery induces blood–brain barrier permeability in GBM patients

To evaluate the impact of surgery after GBM debulking, we analyzed the BBB permeability by MRI in patients with newly diagnosed *IDHwt* GBM. At inclusion, patient median age was 57.0 years (range: 42–73). All the patients benefited from a gross total resection and they all developed local recurrences (Fig. 3A). We evaluated the BBB permeability on MRI images acquired early after surgery (within 48 h after resection; Fig. 3B). All patients presented BBB opening 48 h after complete resection (without remaining enhanced tumor), as revealed by the linear contrast enhancement of the surgical cavity following intravenous gadolinium injection (Fig. 3C and S5). Such openings were thus expected to induce local changes of the cerebral microenvironment in resected GBM patients.

### Characterization of the blood-derived and local myeloid cells following surgery

**Fig. 4:**
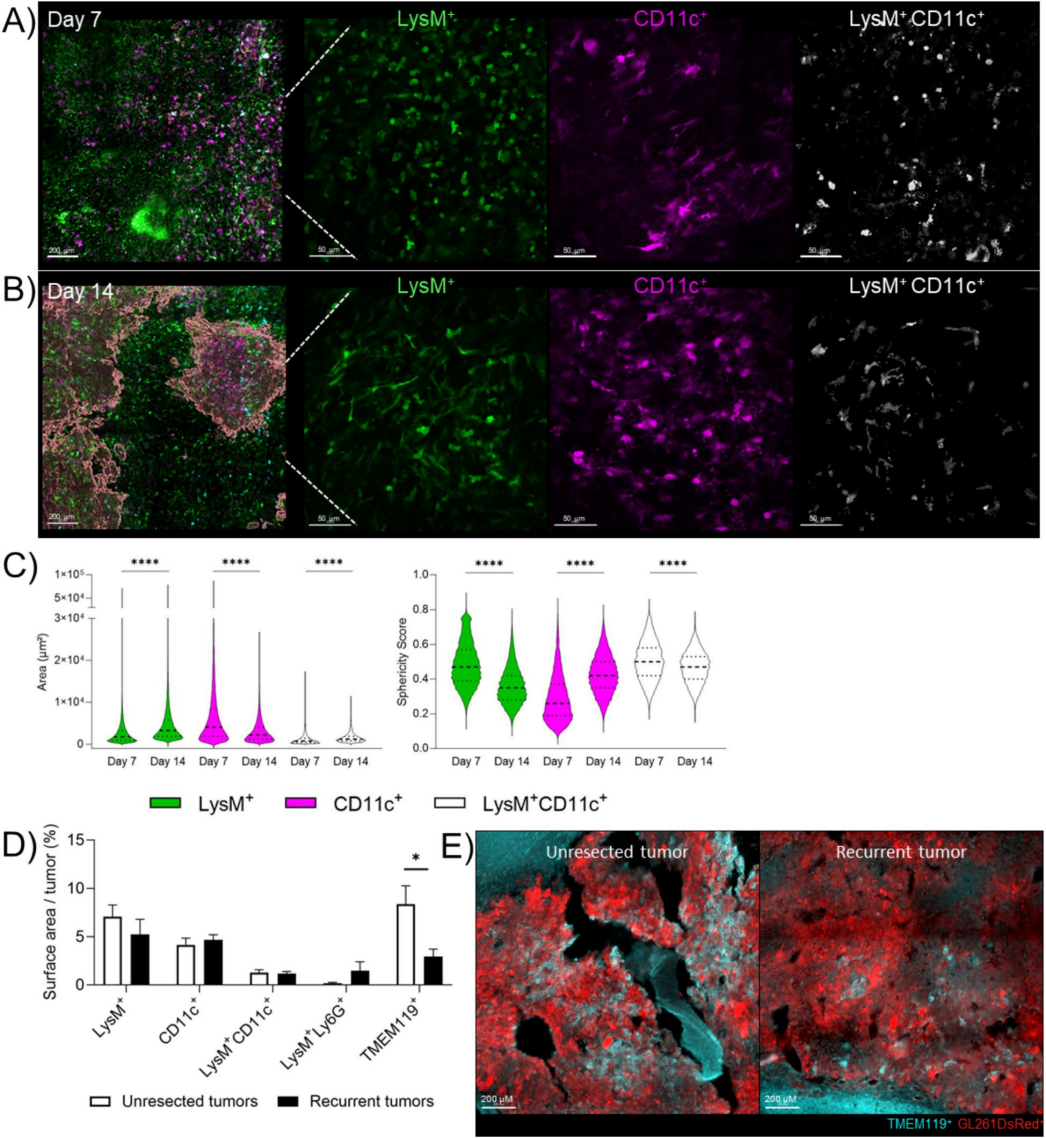
Post-surgical modulation of the brain and tumor microenvironment over time. **A-B**) Two-photon images at day 7 (panel A) and 14 (panel B) post-resection and cranial window implantation showing LysM^+^ cells (green), CD11c^+^ cells (purple), LysM^+^CD11c^+^ cells (white). The reconstruction of the tumor is represented in red in the left panels. Scale bar = 200 µm in the left panels, 50 µm in the three right panels; **C**) Violin plots representing the area (left panel) and sphericity score (right panel) of LysM^+^, CD11c^+^ and LysM^+^CD11c^+^ cells at day 7 and 14. The bar graphs represent mean ± SEM (*n* = 3 animals per group; paired nonparametric Wilcoxon test, ****p < 0.0001); **D**) Percentage of cells expressing LysM^+^, CD11c^+^, LysM^+^CD11c^+^, LysM^+^Ly6G^+^ or TMEM119^+^ in unresected or recurrent tumors at time of sacrifice (*n* = 3–4 animals per group; Mann–Whitney test *p < 0.05); **E** Representative pictures of TMEM119 staining in unresected and recurrent tumors, where tumor cells are GL261-DsRed^+^. Reconstitution, image analyses and cells quantifications were performed using Imaris software

Blood-derived and local myeloid cells are the most abundant immune cells present within GBM [28]. In our mouse model, we have quantified a systemic increase of the blood-derived myeloid cells within the first week following tumor surgery as well as an increase of the BBB permeability during this period in resected animals. Therefore, to determine how the local immunity could be impacted following tumor debulking over time, we used a double transgenic LysM-EGFP/CD11c-EYFP C57BL/6 reporter mice [26] fluorescent for blood-derived myeloid cells and microglia. LysM^+^ cells are monocytes or neutrophils, CD11c^+^ cells are pre-activated microglia and dendritic cells while double LysM^+^/CD11c^+^ cells are monocyte-derived dendritic cells (moDCs) [26]. We used two-photon imaging to analyze morphology and spatial localization of LysM^+^, CD11c^+^ and LysM^+^/CD11c^+^ cells in the resected animals around the resection cavity at day 7, when only a few tumor cells were present (Figure S6A), and at day 14 post-surgery, when recurrent tumors were clearly visible. Indeed, differences in shape and morphology could indicate a transition in the immune response within the SMe, reflecting changes in the polarization and activation state of immune populations over time likely altering their function [46–52]. In unresected animals, the morphology and sphericity of immune cells was comparable to our previous reports [26, 40] (Figure S6B-C). No difference in density of LysM^+^, CD11c^+^, LysM^+^CD11c^+^ cells was found between day 7 and 14 post-surgery (Figure S6C), while modification of their morphology (area and sphericity) was observed in resected animals (Fig. 4A-C). At day 7 post-surgery, LysM^+^ cells assembled in patches, they were smaller and more spherical than at day 14. CD11c^+^ cells, on the contrary, presented a bigger area and a lower sphericity at day 7 compared to day 14. LysM^+^CD11c^+^ cells were smaller and more spherical at day 7 compared to day 14. Altogether, such morphological changes could be early cues of immune environment reorganization between day 7 and 14 post-resection.

Based on these differences in cell morphology during tumor recurrence development, as LyM^+^ cells could be neutrophils or monocytes, and CD11c^+^ cells dendritic cells or microglia, we clarified their phenotypes by immunostaining at the time of sacrifice (Fig. 4D). We used Ly6G staining to identify neutrophils among LysM^+^ cells [26] and TMEM119 [53] staining to discriminate microglia among CD11c^+^ cells. Fluorescent quantification of the brain slices showed no significant difference in the density of LysM^+^, CD11c^+^, LysM^+^CD11c^+^ (moDCs), LysM^+^Ly6G^+^ (neutrophils) cells in the unresected and recurrent tumors. However, TMEM119^+^ cells were decreased ~ 2.8 fold in recurrences meaning that basal microglia are reduced following debulking (Fig. 4E).

### Identification of cellular targets in the microenvironment of recurrent tumors

**Fig. 5:**
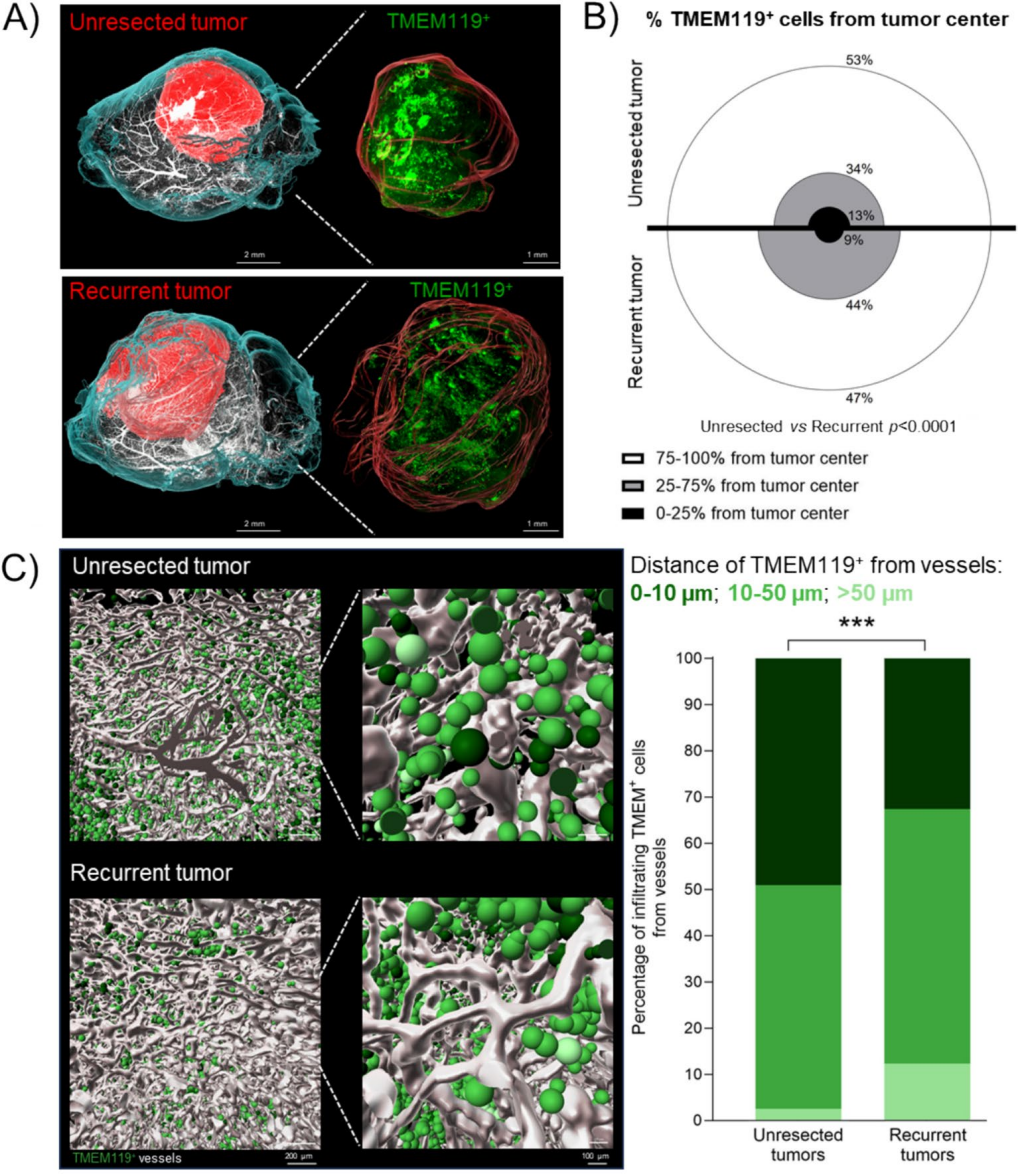
3D spatial characterization of the TMEM^+^ microglia distribution in unresected and recurrent tumors. **A**) Representative 3D light-sheet microscopy images of C57BL/6 mouse brains bearing GBM unresected and recurrent tumors (left and right panel, respectively). The hemi-brains were labeled for: blood vessels (CD31, Podocalyxin and a-SMA in white) and microglia (TMEM119, in green). The reconstruction of the brain and tumor are represented in cyan and red, respectively. Scale bar: 2 mm in left panel, 1 mm in right panel; **B**) Graphical representation of the percentage of TMEM119^+^ microglia from tumor center in each of the distance zone (*n* = 5; Chi^2^ test of independence); **C**) Graphical representations (left panels) and quantification (right panels) of the distance of TMEM119^+^ microglia from vessels (*n* = 5;Chi^2^ test of independence, ****p < 0.0001). Vessels are represented in white, and TMEM119^+^ in different shades of green (darker: 0–10 µm from vessels; medium: 10–50 µm; lighter: > 50 µm). Scale bar: 200 µm in each left panel, 100 µm in each right panel. Reconstitution and image analyses were performed by using Imaris software

To gain a deeper understanding of microglial cells, we examined their spatial distribution throughout the entire tumor at day 14 post-surgery and/or cranial window implantation. At this time point all mice, both unresected and resected, were alive without clinical symptoms and had tumors of comparable size (Figure S1C). We conducted whole mount staining of TMEM119^+^ cells and blood vessels, followed by light sheet imaging and 3D image reconstruction. We then performed a cumulative analysis of the TMEM119^+^ cells using hierarchical clustering to represent the distance of these cells from the tumor center in both unresected and resected tumors (Fig. 5A-B). For this analysis, tumors were divided into 3 distance zones from the tumor center: tumor center (0–25% from the center of the tumor), infiltrative zone (25–75% from the center of the tumor), tumor periphery (75–100% from the center of the tumor). As shown in Fig. 5B, less TMEM119^+^ cells were localized in the tumor periphery and tumor center in recurrent tumors compared to unresected tumors (47% *vs* 53% and 9% *vs* 13%, respectively) while more were localized in the infiltrative zone (44% *vs* 34%). These findings show that in the SMe, more microglia were found close to the tumor center and less in the tumor periphery in comparison with unresected tumors. As immune cells can use vessels as a route of migration to the tumor, we then evaluated the distance of TMEM119^+^ cells from blood vessels within the tumor (Fig. 5C-D). Our results showed that in the unresected tumors 49% of TMEM119^+^ cells are in contact with blood vessels (measured as distance < 10 μm) while 2.5% are distant from blood vessels (> 50 µm). By comparison, in recurrent tumors, 33% of TMEM119^+^ are in proximity of blood vessels while 12% are distant. These results show that, in recurrences, TMEM119^+^ microglial cells have more tendency to diffuse from tumor periphery and perivascular spaces to the tumor core as compared to unresected tumors.

In addition, TMEM119^+^ cells were significantly more spherical in recurrent tumors in comparison with unresected tumors (Figure S7). These TMEM119^+^ cells could also represent a proportion of CD11c^+^ spherical microglia previously observed by biphotonic imaging (Fig. 4A-C). All these changes in TMEM119^+^ cells quantity, organization and morphology might reflect changes in cell activation status [54].

**Fig. 6:**
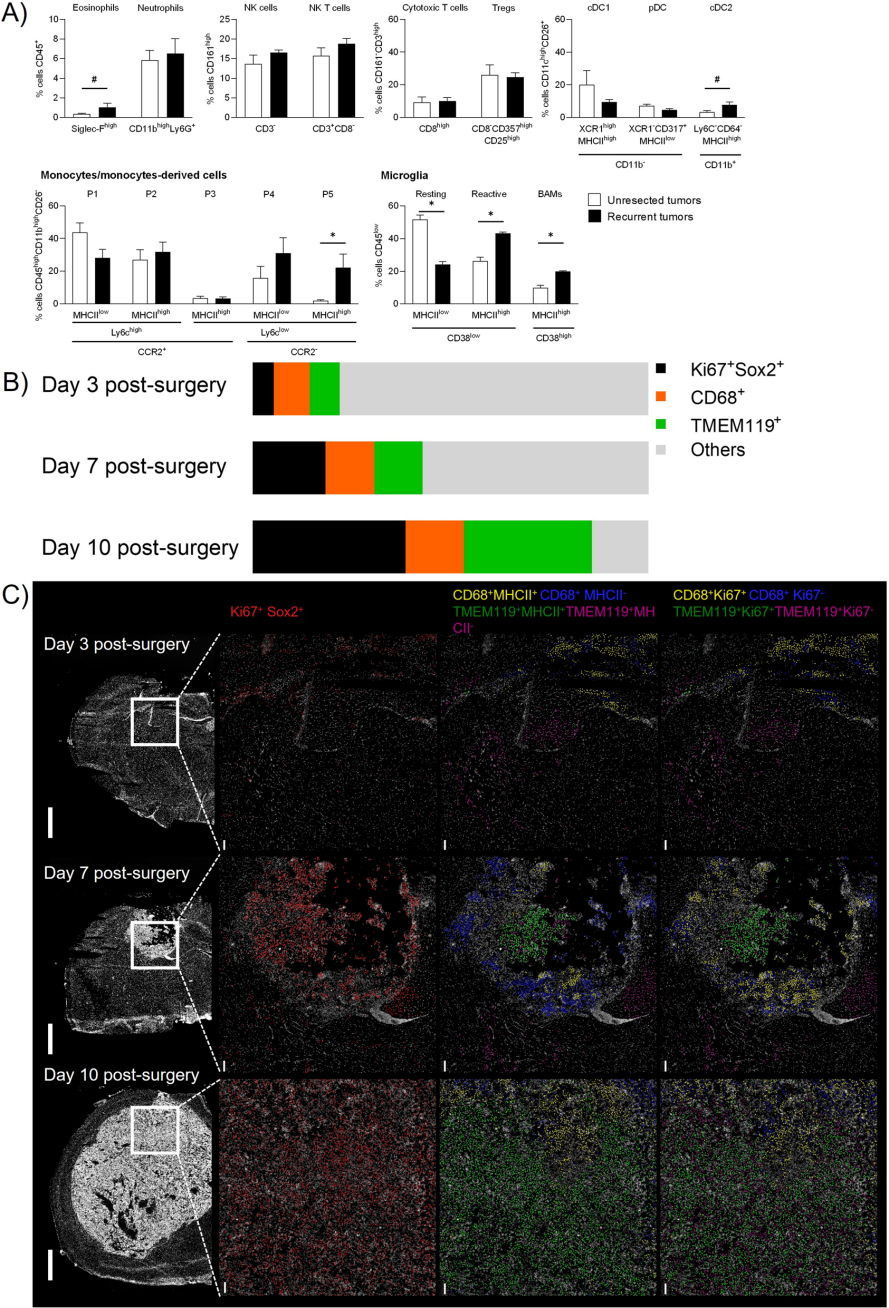
The immune landscape of unresected and recurrent tumors and the SMe. **A**) Multiparametric flow cytometry analysis of tumors using the gating strategy reported in Figure S8 (DC: dendritic cells; BAMs: border associated macrophages). Statistical analysis was performed using nonparametric Mann–Whitney test (mean ± SEM, *n* = 4–5; ^#^*p* = 0.05, *p < 0.05); **B**) Percent of Ki67^+^Sox2^+^, CD68^+^, TMEM119^+^ among the total cells quantified in the region of interest of hyperplexed immunofluorescence images during GL261 recurrence development at day 3, 7, 10 post-surgery (average value from 3 different slices); **C**) Representative images of post surgical resection cavity in GL261-DsRed bearing animals imaged with MACSima technology during recurrence development at day 3, 7 and 10 post surgery (left panels, white DAPI staining). The right panels show the spatial location of Ki67^+^Sox2^+^ (middle left panel), CD68^+^MHCII^+^, CD68^+^MHCII^−^, TMEM119^+^MHCII^+^, TMEM119^+^MHCII^−^ cells (middle right panel) and CD68^+^Ki67^+^,CD68^+^ Ki67^−^, TMEM119^+^Ki67^+^, TMEM119^+^Ki67^−^ (right panel). Scale bar: 1 mm in left panel, 100 µm in the other panels

To investigate microglial cells activation in recurrent tumors, we developed a flow cytometry panel capable of distinguishing between myeloid and lymphoid subpopulations (Figure S8-S9). Thanks to this panel and gating strategy, we were able to discriminate between monocytes (P1) and their differentiated cells (moDC: P2 and P3; monocyte-derived macrophages: P4 and P5, related to anti-inflammatory macrophages), as well as resting and reactive microglia, and border-associated macrophages (BAMs). Results showed that the number of anti-inflammatory P5 macrophages significantly increased in post-surgical recurrences compared to unresected tumors (Fig. 6A). In parallel, resting microglia decreased while reactive microglia and BAM increased in recurrent tumors. Eosinophils and dendritic cells cDC2 also showed a tendency to increase in number. Altogether these results revealed that anti-inflammatory macrophages, reactive microglia and BAMs are the main immune cells enriched in recurrent tumors.

This was also confirmed in the GBM9 model (Figure S2). Indeed, Iba1 staining was performed to identify macrophage/microglia cells. In both unresected and resected tumors, in the infiltrative tumor zone we observed a mix of ramified and amoeboid Iba1^+^ cells. In resected tumors, in the identifiable dense tumor mass, Iba1^+^ cells were mainly amoeboid reflecting their activated phenotype. Anti-inflammatory ARG-1^+^ cells were instead observed in highly proliferative zones while they were mainly absent in the infiltrative areas. These results support that the balance between activated microglia and anti-inflammatory macrophages plays a key role in tumor recurrences.

To study the dynamic of recruitment of these cells, we performed spatial multiplexed immunofluorescences at day 3, 7 and 10 post-surgery (Fig. 6B and S10A). Galectine-3 antibody was used to discriminate bone marrow-derived cells from microglia cells, microglia being Galectine-3^−^ [55]. Firstly, we have quantified the proportion of proliferative tumor cells (Sox2^+^ Ki67^+^) present in or around the resection cavity borders where we also quantified blood-derived macrophages CD68^+^ Galectine-3^+^ and microglia cells TMEM119^+^Galectine-3^−^. Tumor cells increased from 5% (day 3), to 18% (day 7), to 39% (day 10) confirming tumor regrowth during this time window. At day 3 and day 7, the proportion of blood-derived macrophages (day 3: 9%; day 7: 12%) and microglia (day 3: 8%; day 7: 12%) was similar. At day 10, the proportion was blood-derived macrophages remained similar (15%) while microglia increased massively (32%). Until day 7, we could observe a spatial segregation between blood-derived macrophages and microglia, blood-derived macrophages being located at the tumor periphery and microglia closer to the tumor center. This spatial repartition was lost at day 10. In order to gain insights in their state of activation, we subdivided blood-derived macrophages and microglia in proliferative cells Ki67^+^ and antigen-presenting cells MHCII^+^ (Fig. 6C and S10B). For CD68^+^ macrophages, no clear pattern of activation was observed between the different timings and markers analyzed at the exception of a massive quantity of CD68^+^MHCII^−^ cells at day 7. For TMEM119^+^ microglia, an increase of the proportion of TMEM119^+^MHCII^+^ and TMEM119^+^Ki67^+^ was quantified during recurrence development. At day 7, the TMEM119^+^Ki67^+^ cells were located inside the recurrent tumors while the TMEM119^+^Ki67^−^ were outside the recurrent tumors. The TMEM119^+^MHCII^+^ cells were located inside the recurrent tumors while the TMEM119^+^MHCII^−^ were both inside and outside the recurrent tumors.

Altogether, these results revealed that activated blood-derived macrophages and microglia are present since the onset of recurrence and all along its development.

### Tailoring a combinatory regimen to tackle the immune landscape of the post-surgical microenvironment

Based on our longitudinal characterization of the SMe, we hypothesized that limiting the accumulation of anti-inflammatory macrophages and promoting anti-tumor reactive microglia directly after surgery, we could improve mouse survival. We thus combined the GemC_12_-LNC (which has both anticancer properties [56] and immunostimulatory capacity [57]) with the SMAC mimetic compound GDC-0152. GDC-0152 is a small-molecule antagonist of inhibitor of apoptosis proteins (IAP) which has both pro-apoptotic properties [38, 58, 59] and immunomodulatory properties characterized by the decrease of pro-tumoral P5 macrophages and the promotion of anti-tumoral microglia with APC function in primary GBM tumors [40]. This molecule is well tolerated and able to pass the BBB in physiological condition, but it could be administered systemically after surgery to benefit from BBB opening to target the systemic immune response more efficiently.

**Fig. 7:**
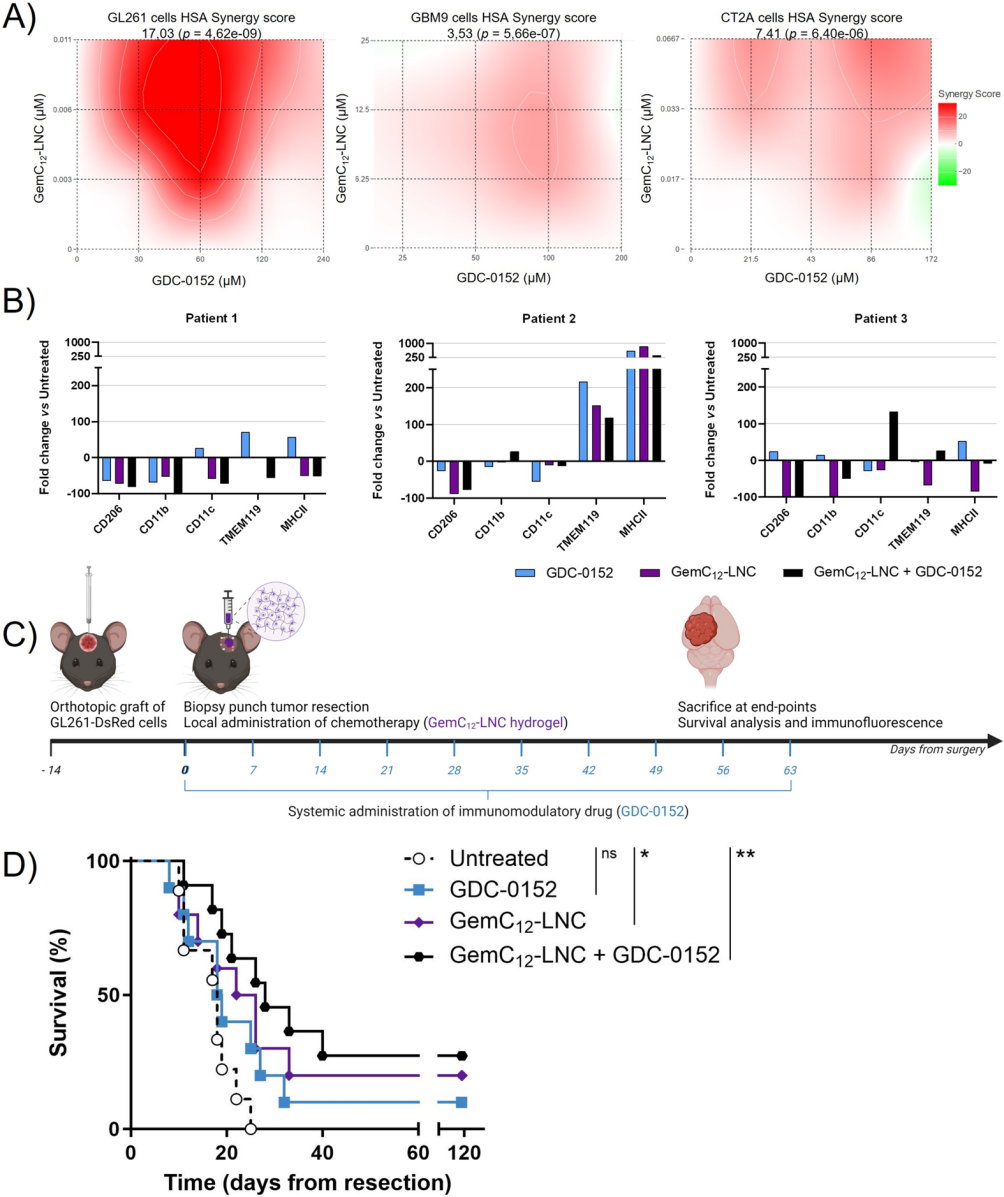
A tailored combinatory approach to target the SMe and delay the onset of GBM recurrences. **A**) Heat maps representing the HSA synergy score obtained using a 3 × 4 matrix to test the GemC_12_-LNC and GDC-0152 combination on GL261 (left panel), GBM9 (middle panel) and CT2A cells (right panel); **B**) Effect of GDC-0152, GemC_12_-LNC and their combination on the immune cells expression of CD206, CD11b, CD11c, TMEM119, MHCII in tumoroids from three different patients as fold change versus untreated tumoroids; **C)** Schematic representation of the treatment regimen proposed to target the SMe and the post-mortem analysis performed on the extracted brains; **D**) Kaplan–Meier survival curves of the tumor-bearing C57BL/6 mice receiving tumor surgery and treatment at day 14 post-grafting (*n* = 8–9; ns = non-significant; *p < 0.05; **p < 0.01)

Such drug combination was first validated in GL261, GBM9 and CT2A cell lines to eliminate the possibility of antagonistic interactions between the drugs in GBM cells. The treatment with GemC_12_-LNC and GDC-0152 exhibited overall HSA synergy scores of 17.03 for GL261 cells, 3.53 for GBM9 cells and 7.41 for CT2A cells, indicating a synergistic or additive pharmacological interaction (Fig. 7A).

To evaluate the effect of the combination on the immune cells associated to human GBM, we used tumoroid human-derived model preserving GBM heterogeneity [44]. Tumoroids derived from three patients were treated with the single drugs and with a combination of both for 72 h. At the end of the treatment, flow cytometry analysis was performed to quantify the densities of anti-inflammatory cells CD206^+^, myeloid cells CD11b^+^ and CD11c^+^, basal microglia TMEM119^+^ and antigen-presenting cells MHCII^+^ (Fig. 7B). We observed that CD11b and CD11c varied between each patient and condition. CD206 was decreased in each patient and condition except for GDC-0152 treatment of patient 3. TMEM119 increased in each condition in patient 2 and decreased in each condition in patients 1 and 3 except for the combo of the patient 3. MHCII increased in each condition in patient 2 and in the GDC-0152 condition of patients 1 and 3. These results confirmed previously results obtained with GDC-0152 alone [40] and suggest that these drugs and the combo can modulate the GBM immune landscape toward a less anti-inflammatory phenotype. To confirm the efficacy of the combination, we moved forward to in vivo studies. To exploit the GemC_12_-LNC hydrogel properties and the ability of GDC-0152 to pass the BBB, we administrated GemC_12_-LNC locally in the tumor resection cavity followed by weekly systemic administration of GDC-0152 from the time of surgery (Fig. 7C). While in monotherapy only GemC_12_-LNC significantly delayed the death of animals (32 days untreated *vs* 32.5 days GDC-0152 *p* = 0.1512; 32 days untreated *vs* 38 days GemC_12_-LNC *p* = 0.0490), the combinatory treatment further increased the median survival (32 days untreated *vs* 42 days GemC_12_-LNC + GDC-0152 32 *p* = 0.0027) (Fig. 7D). Three animals were long-term survivals (lived longer than 120 days) in this group and their brains did not show presence of malignant tumor cells post-mortem (*data not shown*).

**Fig. 8:**
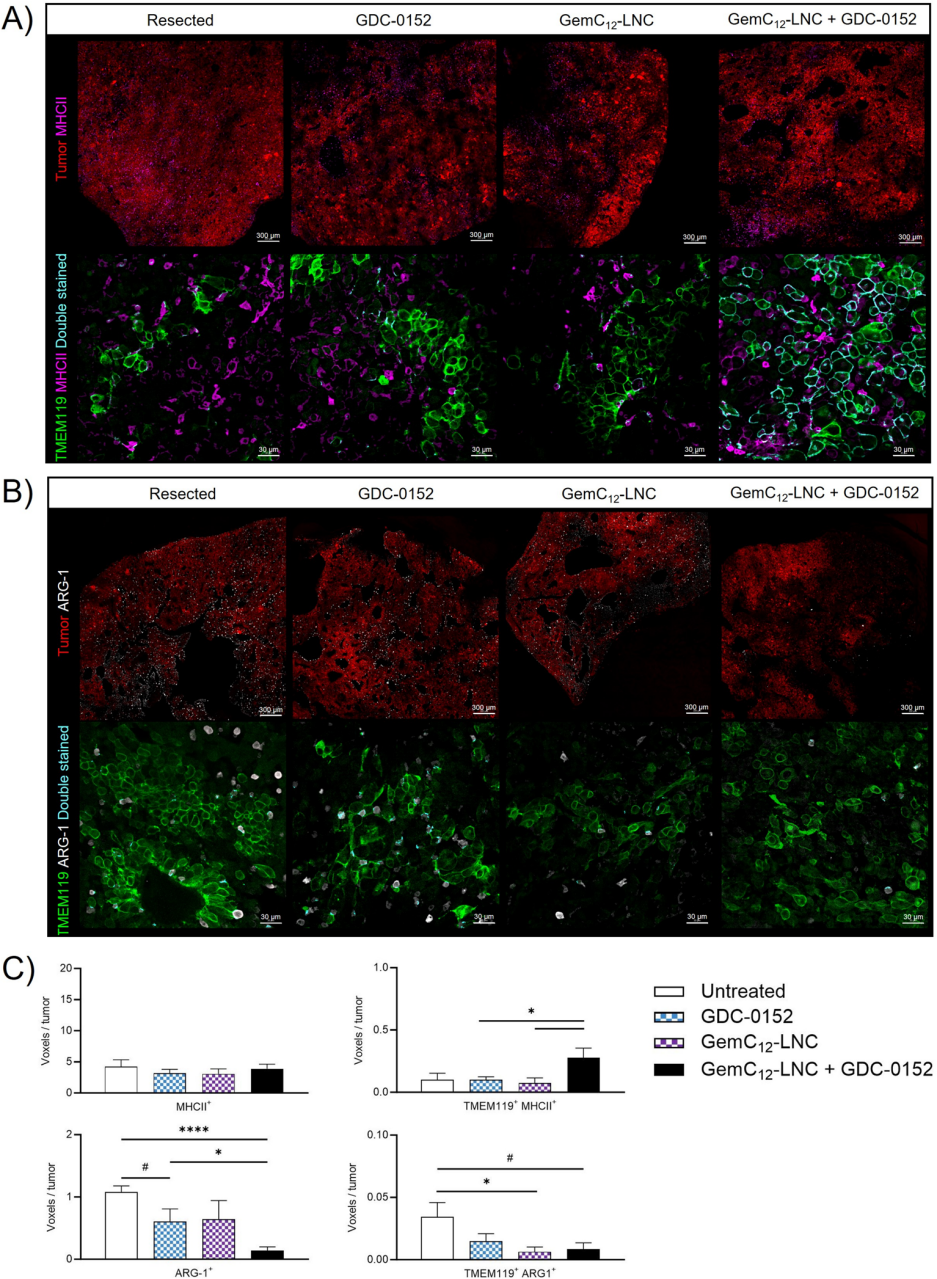
A combinatory approach to reverse the post-surgical immunity. **A-B**) Representative confocal microscopy images of coronal brain slices from animals who had received resection or resection plus treatment(s). Sections were immuno-stained against MHCII (lower panel A, in purple), ARG-1 (lower panel B, in white) and TMEM119 (lower panels A and B, in green) while tumor cells transgenetically expressed DsRed (in red in the upper panels A and B). MHCII^+^/TMEM119^+^ cells (panel A) and ARG-1^+^/TMEM119^+^ (panel B) are represented in cyan in the lower panels to evaluate the level of microglial activation and the anti-inflammatory status, respectively. Scale bar: 300 µm for upper panels, 30 µm for lower panels; **C**) Quantifications of the densities of each cell sub-population (mean ± SEM, *n* = 4; Unpaired t test, ^#^ 0.05 < *p* < 0.06; *p < 0.05; ****p < 0.0001)

The brain of animals that were sacrificed when reaching the clinical endpoints presented recurrent tumors and were used to characterize the impact of the different treatments on the tumor microenvironment, with specific focus on microglial reactivity and anti-inflammatory states (Fig. 8A-B). The proportions of TMEM119^+^ cells upregulating MHCII or ARG-1 were respectively used as indexes of activation and anti-inflammatory phenotypes [40]. The combinatory treatment increased the amount of activated microglia with APC function (TMEM119^+^MHCII^+^ cells) compared to the monotherapies (*vs* GDC-0152 *p* = 0.0459, *vs* GemC_12_-LNC *p* = 0.0354; Fig. 8C) while significantly reducing the number of anti-inflammatory cells (ARG-1^+^ cells) compared to the untreated and GDC-0152 treated animals (*p* < 0.0001 and *p* = 0.0452, respectively). The number of anti-inflammatory microglia (TMEM119^+^ARG-1^+^ cells) was similarly reduced in the GemC_12_-LNC group compared to the untreated control (*p* = 0.0351) and close to significance for the combination group (*p* = 0.0565). Taken together, our results demonstrate that the proposed combinatory regimen increased the total number of APC presenting reactive microglia in the tumor microenvironment while decreasing all anti-inflammatory cell populations.

## Discussion

Maximal safe resection is the mainstay of GBM treatment, necessary to establish a diagnosis (through histopathological analysis of the resected tissue) and the improving prognosis while preserving patient functional activity. Even though neurosurgical techniques have advanced in the last decades and gross total resection is now considered a safe procedure [60, 61], the data concerning its impact on tumor recurrences are still limited. Our gadolinium-enhanced MRI data in GBM patients suggest that BBB is compromised in proximity of the surgical margins where cerebral microenvironment is changed. BBB leakage has already been observed in stroke and traumatic brain injury patients [62, 63]. Although it leads to cerebral flow alteration, cerebral metabolism imbalance, oedema and accumulation of inflammatory molecules, it can also be seen as an interesting window for systemic therapeutic intervention [64]. Because longitudinal follow-up of the local immune landscape following surgery is impossible to perform in human patients with GBM, we used an orthotopic GBM mouse model to characterize the impact of surgery on tumor relapse over time via different imaging and analytical techniques. Immunocompetent murine models are effective for generating tumors that resemble human GBM, making them well-suited for investigating immune responses within the tumor microenvironment and for studying their interactions with the tumor stroma [65, 66]. We were able to establish a tumor resection model compatible with several type of analyses after a cranial window implantation. For this study, we selected the GL261 syngeneic mouse model, a model that we have mastered and extensively studied [26, 27, 32, 40], to analyze the temporal modifications of the brain and tumor microenvironment from surgery to recurrence [65, 67]. As already observed, in our model, tumor resection improved mice survival but we also observed a fast regrowth of post-surgical tumors supporting the hypothesis of a deleterious debulking-induced environment [13, 31, 33]. We indeed observed a rise in specific immune cell populations in the blood up to day 10. Concurrently, at that time point, we also observed the closure of the BBB. Besides potentially increasing the recruitment of immune cells to the tumor, this temporary permeabilization of the BBB could offer a unique opportunity for the efficient systemic delivery of therapeutic drugs. In line with these results, we have previously modulated the post-surgical immune responses by administering ibuprofen systemically directly after surgery in combination with local chemotherapy showing a better therapeutic outcome [16]. Before us, a few studies had evaluated the immune landscape of brains after surgery in mice models, in the context of the development of immunotherapies. A reduction in blood lymphocytes was observed by Otvos *et* al. 7 days post-resection in GL261 and CT2A models, mainly explained by a strong reduction in circulating T cells (CD4^+^ and CD8^+^ T cells) and despite a significant increase in circulating granulocytic myeloid-derived suppressor cells (MDSC) [33].

Our phenotypic and imaging characterization evidenced that recurrent tumors were particularly enriched in anti-inflammatory macrophages, BAMs and reactive microglia. Higher percentage of suppressive M2 TAMs and CD4^+^Foxp3^+^ Tregs were also observed by Predina *et* al. in recurrent tumors of lung cancers compared to primary tumors of equivalent size [68]. Knudsen et al*.* demonstrated that post-surgical infiltrating microglia/macrophages can induce GBM cells proliferation and upregulation of several stem cell related genes in recurrent tumors, suggesting their role in promoting tumor recurrences [5]. In the CT2A mouse model, Choi *et* al. have instead shown that tumor debulking resulted in a reduction of MDSC along with the recruitment of effector T lymphocytes (CD4/CD8 T cells) into the resection area at 2, 4 and 6 days post-surgery and increased dendritic cells at day 4 post-surgery compared to unresected GBM-tumors bearing animals [17]. Mass cytometry (CyTOF) further highlighted that proportions of resident macrophages and resting microglia were lowered while siglecF^+^ macrophages and activated microglia increased as well as CD4^+^ and CD8^+^ T cells. Removal of the tumor core thus decreased the number of immunosuppressive immune cells, but increased the recruitment of newly activated immune cells [30] and reactive resident brain cells promoting tumor cells migration and proliferation [5, 19]. Ortiz Rivera et al*.* have shown that GBM recurrent tumors in mice had altered tumor-infiltrating myeloid cells polarization—shifting from a CD206^+^/CD86^−^ to a CD206^+^/CD86^+^ profile between day 7 and 14 post-surgery—with significant increases of pro-tumorigenic cytokines (IL4, IL5, IL10, IL12, IL17, vascular endothelial growth factor VEGF, and monocyte chemoattractant protein 1 MCP1/CCL2) compared to primary tumors [69]. The recurrent tumors also showed an upregulation of phosphorylated proline-rich tyrosine kinase (Pyk2) and focal adhesion kinase (FAK) without differences in the total protein expression [70]. Our longitudinal study of the SMe on the same animals from surgery to recurrence confirmed that the dynamic changes in the SMe are dependent on timing. This dynamic changes were accompanied by a spatial clustering of activated microglia and macrophages different than the one described in primary tumors [71]. We identified that the second post-surgical week as the period during which tumor cell growth is boosted to form recurrences, and revealing that the recruited immune cell subtypes in the SMe could contribute to the growth promoting environment and could therefore serve as potential therapeutic targets.

In the current clinical practice, systemic chemotherapy and radiotherapy are not started immediately after GBM debulking surgery as they might compromise the recovery of patient post-surgery and have a negative impact on the wound healing process [72, 73]. This practice might be reconsidered knowing that 90% of GBM recurrences arise along the post-surgical cavity borders and that tailored strategies focally targeting the SMe have been largely unexplored to avoid GBM relapse. Understanding the interactions between immune and residual tumor cells, their morphologies and local distribution around the resection cavity or within recurrent tumors should help designing tailored drug delivery systems to normalize the SMe. Here we have shown that blood vessels are strongly impacted by surgery, which could be an advantage for the accumulation of drug-loaded nanoparticles around residual tumor cells of the SMe. Our experience of local nanomedicine-based GBM treatments along with this imaging based characterization of recurrent tumors environment, guided us to combine the local GemC_12_-LNC treatment with a systemic administration of the SMAC mimetic GDC-0152, a pro-apoptotic drug with immunomodulatory properties. SMAC mimetics antagonize the IAPs, which are known regulators of cell death that contribute to treatment resistance in several cancers including GBM [74]. We have previously found that systemic treatment with the small molecule GDC-0152 potentiates immune cell infiltration and the remodeling of tumor vasculature toward slower tumor growth and increased survival of GBM-bearing mice [38, 40]. We have shown that GDC-0152 acts by decreasing anti-inflammatory macrophages, and driving microglia toward an anti-tumoral phenotype via caspase-3 pro-inflammatory activation and TNFα signaling [40]. In addition, we were also able to demonstrate that GemC_12_-LNC alone could impact the GBM immune environment, as previously demonstrated in lymphoma and melanoma-bearing mice [75]. As post-surgical recurrences show a higher proportion of activated microglia, its reprogramming by immediate GDC-0152 treatment from the day of surgery was expected to have a beneficial role in the prevention of recurrences especially when combined to the weakening of residual tumor cells by GemC_12_-LNC. Our results indeed show a synergistic effect to delay the recurrences in vivo providing long-term recovery for some animals. The combination of the two active agents induced an increased expression of MHCII and decreased expression of ARG-1 in microglial cells which can be seen as a proof-of-concept that tailored treatment targeting the SMe can be effective to prevent post-surgical recurrences in the long-term.

## Conclusion

This study demonstrates that surgery induces both systemic and local physiological changes in GBM-bearing mice. Through longitudinal characterization of the SMe from tumor surgery to recurrence, we identified immune cells within recurrences that could serve as therapeutic targets. Additionally, we observed a transient increase in BBB permeability post-surgery, which offers a therapeutic window for the effective systemic delivery of immunomodulatory and anti-inflammatory agents. We also proposed and validated a combined treatment regimen involving local administration of a chemotherapeutic hydrogel and systemic delivery of an immunomodulatory drug, which delayed recurrence and improved survival. These findings highlight the importance of targeting not only residual tumor cells post-surgery but also protumoral macrophages, activated microglia, and BAMs, as these cells likely play a critical role in recurrence onset.

## Supplementary Information

Supplementary Material 1.

## Data Availability

The datasets used and/or analyzed during the current study are available from the corresponding authors on reasonable request.

## Abbreviations

APCs: Antigen-presenting cells (APCs)
ARG-1: Anti-Arginase 1 (ARG-1)
BAMs: Border-associated macrophages
BBB: Blood–brain barrier
BSA: Bovine Serum Albumin
DMSO: Dimethyl sulfoxide
DPBS: Dulbecco’s Phosphate buffered saline
GBM: Glioblastoma
GemC_12_-LNC: Lauroyl-gemcitabine lipid nanocapsules
KPS: Karnofsky Performance Status
MRI: Magnetic resonance imaging
PBS: Phosphate-buffered saline
PFA: Paraformaldehyde
TAMs: Tumor associated macrophages/microglia
SMe: Post-surgical microenvironment
SPECT: Single-Photon Emission Computed Tomography
