# Supplementary material for: Tailoring glioblastoma treatment based on longitudinal analysis of post-surgical tumor microenvironment
Source: J Exp Clin Cancer Res. 2024 Nov 28;43:311. doi: 10.1186/s13046-024-03231-4 (PMC11603899; doi:10.1186/s13046-024-03231-4)
Supplement: Supplementary file 1 — Supplementary Material 1. [file 13046_2024_3231_MOESM1_ESM.docx]

Tailoring glioblastoma treatment based on longitudinal analysis of post-surgical tumor microenvironment

Chiara Bastiancich^1,2,3,4*^, Emmanuel Snacel-Fazy^1^, Samantha Fernandez^5^, Stéphane Robert^6^, Roberta Stacchini^1^, Léa Plantureux^6^, Sébastien Boissonneau^7,8^, Benoit Testud^9,10,11^, Benjamin Guillet^5,6,12^, Franck Debarbieux^13,5,14^, Hervé Luche^15^, Dominique Figarella-Branger^1^, Marie-Anne Estève^1,16^, Emeline Tabouret^1,17,18^, Aurélie Tchoghandjian^1,4*^

# Supplementary material

**Table S1.** Flow cytometry panel for blood immunophenotyping

| **Antibody**  **– conjugated fluorophore** | **Concentration used** | **Provider** | **Reference number** |
| --- | --- | --- | --- |
| Viakrome viability dye – 808 | 2.5 µL per tube | Beckman Coulter | C36628 |
| CD45 – BB700 | 2.5 µg/mL | BD | 566440 |
| CD11b – BV421 | 0.625 µg/mL | Biolegend | 101235 |
| CD11c – BUV496 | 0.625 µg/mL | BD | 750450 |
| CD8a – BV650 | 0.625 µg/mL | Biolegend | 100741 |
| CD161 – PE/Cy5 | 2.5 µg/mL | Biolegend | 108715 |
| CD357 (GITR) – APC | 0.156 µg/mL | Biolegend | 126311 |
| Ly6G – FITC | 3.125 µg/mL | BD | 561105 |
| SiglecF – BV605 | 0.625 µg/mL | BD | 740388 |

**Table S2.** Antibodies used for brain clearing and immunostaining

| **Antibody** | | **Dilution used** | **Provider** | **Reference number** |
| --- | --- | --- | --- | --- |
| Primary | α-SMA | 1/500 | Abcam | ab21027 |
|  | CD31 | 1/500 | R&D systems | AF3628 |
|  | CD45 | 1/200 | Santa Cruz | sc-53665 |
|  | GFAP-VioR667 | 1/50 | Miltenyi Biotec | 130-131-151 |
|  | Podocalyxin | 1/500 | R&D systems | AF1556 |
|  | TMEM119 | 1/700 | Abcam | Ab209064 |
| Secondary | Anti-rabbit AF488 | 1/600 | Invitrogen | A11055 |
|  | Anti-goat AF647 | 1/600 | Invitrogen | A21447 |
|  | Anti-rabbit AF488 | 1/600 | Molecular Probes | A11008 |
|  | Anti-rat AF647 | 1/600 | Jackson ImmunoResearch | 712-605-150 |
|  | Anti-rabbit AF555 | 1/600 | Invitrogen | A31572 |

**Table S3.** Flow cytometry panel for brain immunophenotyping

| **Antibody**  **– conjugated fluorophore** | **Concentration used** | **Provider** | **Reference number** |
| --- | --- | --- | --- |
| Viakrome viability dye – 808 | 2.5 µl per tube | Beckman Coulter | C36628 |
| CD45 – BB700 | 2.5 µg/mL | BD | 566440 |
| CD11b – BV421 | 0.625 µg/mL | Biolegend | 101235 |
| CD11c – BUV496 | 0.625 µg/mL | BD | 750450 |
| Ly6C – PE/Cy7 | 0.3 µg/mL | Biolegend | 128017 |
| CD8a – BV650 | 0.625 µg/mL | Biolegend | 100741 |
| MHCII – APC/Cy7 | 0.3125 µg/mL | Biolegend | 107627 |
| CD161 – PE/Cy5 | 2.5 µg/mL | Biolegend | 108715 |
| CCR2 –AF594 | 0.25 µg/mL | R&D systems | FAB5538T |
| CD357 (GITR) – APC | 0.156 µg/mL | Biolegend | 126311 |
| CD317 – AF700 | 2.5 µg/mL | Biolegend | 127037 |
| Ly6G – FITC | 3.125 µg/mL | BD | 561105 |
| SiglecF – BV605 | 0.625 µg/mL | BD | 740388 |
| XCR1 – BV650 | 1.25 µg/mL | Biolegend | 148220 |
| CD38 – BUV737 | 5 µg/mL | BD | 741748 |
| CD44 – BV510 | 5 µg/mL | Biolegend | 103043 |
| CD3 – FITC | 3.125 µg/mL | Biolegend | 100203 |
| CD64 – BV786 | 10 µg/mL | BD | 741024 |
| CD26 – PE | 0.625 µg/mL | Biolegend | 137803 |
| CD25 – BUV395 | 10 µg/mL | BD | 564022 |

**Table S4.** Spatial hyperplexed immunofluorescence imaging panel

| **Antibody**  **– conjugated fluorophore** | **Dilution used** | **Provider** | **Reference number** |
| --- | --- | --- | --- |
| CD68 – FITC | 1/50 | Miltenyi Biotec | 130-112-855 |
| MHCII – FITC | 1/50 | Miltenyi Biotec | 130-112-386 |
| TMEM119 – PE | 1/50 | Cell signaling | 90840 |
| Sox2 – FITC | 1/50 | Miltenyi Biotec | 130-120-790 |
| Ki67 – FITC | 1/50 | Miltenyi Biotec | 130-117-691 |
| Galectin-3-FITC | 1/50 | Miltenyi Biotec | 130-118-647 |

**Table S5.** Flow cytometry panel for tumoroid immunophenotyping

| **Antibody**  **– conjugated fluorophore** | **Dilution used** | **Provider** | **Reference number** |
| --- | --- | --- | --- |
| Anti-CD206 – PE | 1/50 | Miltenyi Biotec | 130-124-233 |
| Anti-CD11b - PerCP-Vio 700 | 1/50 | Miltenyi Biotec | 130-110-557 |
| Anti-CD11c - PEVio770 | 1/50 | Miltenyi Biotec | 130-113-588 |
| Anti-TMEM119 - AF647 | 1/100 | Abcam | Ab225494 |
| Anti-IA/IE (MHCII) - APC/Cyanine7 | 1/100 | Biolegend | 107627 |

**Patient-derived** **GBM9 xenograft orthotopic resection model**

The experiments involving grafting of NMRI nude mice with GBM9 cells were performed in Belgium following the Belgian national regulation guidelines and were approved by the ethical committee for animal care of the health science sector of the Université Catholique de Louvain (2019/UCL/MD/004). Mice were acquired by the authorized provider Janvier at the age of 4 weeks and housed in enriched cages placed in a temperature- and hygrometry-controlled room. They had free access to water and food and were monitored daily. After one week of acclimatation, animals were anesthetized by ketamine/xylazine (100 and 10 mg/kg intraperitoneal injection, respectively) and fixed in a stereotactic frame. The skin surface on the head was disinfected by application of an antiseptic solution (Vétédine® solution, Vetoquinol, Lure, France) and lidocaine (10 mg/mL; Aguettant, Lyon, France) was injected subcutaneously at the site of incision. The eyes were protected with an ophthalmic gel (Ocry-gel, TVM lab, Lempdes, France). An incision was made along the midline and a burr hole was drilled into the skull at the parietal lobe (0.5 mm posterior and 2.1 lateral to the bregma) using a high-speed drill. A 10 μL 26 s gauge syringe with cemented 51 mm needle was used to inject 5×10^5^ GBM9 cells in the cortex (2.5 mm deep from the outer border of the brain), using an automatic pump device at a speed of 0.7 μL/min. The wound was then closed using a tissue adhesive glue (3M Vetbond®, Sergy-Pontoise, France) and the animals recovered under an infrared heating lamp. Seventy-six days post-grafting, animals were either left untreated or tumor resection was performed using the biopsy punch technique previously described in the manuscript text covering the dural window with a Neuro-Patch^®^ (Aesculap, Germany) impregnated with fibrin sealant (Tisseel Prima; Baxter, France). Mice were then monitored daily, and their body weight was measured every week. As *in vivo* tumor growth in this model is very slow compared to typical GBM murine models (e.g. GL261, U-87, CT2A) and leads to diffused tumors difficult to be observed by imaging, a biopsy punch was performed at the cell injection site when the first animals started to die due to clinical symptoms. The remaining animals were divided in two groups, and were either left unresected or received resection (same procedure described for GL261-DsRed). The mice were sacrificed when they reached the endpoints or at day 283 post-grafting for histological analysis.

**Supplementary figures**


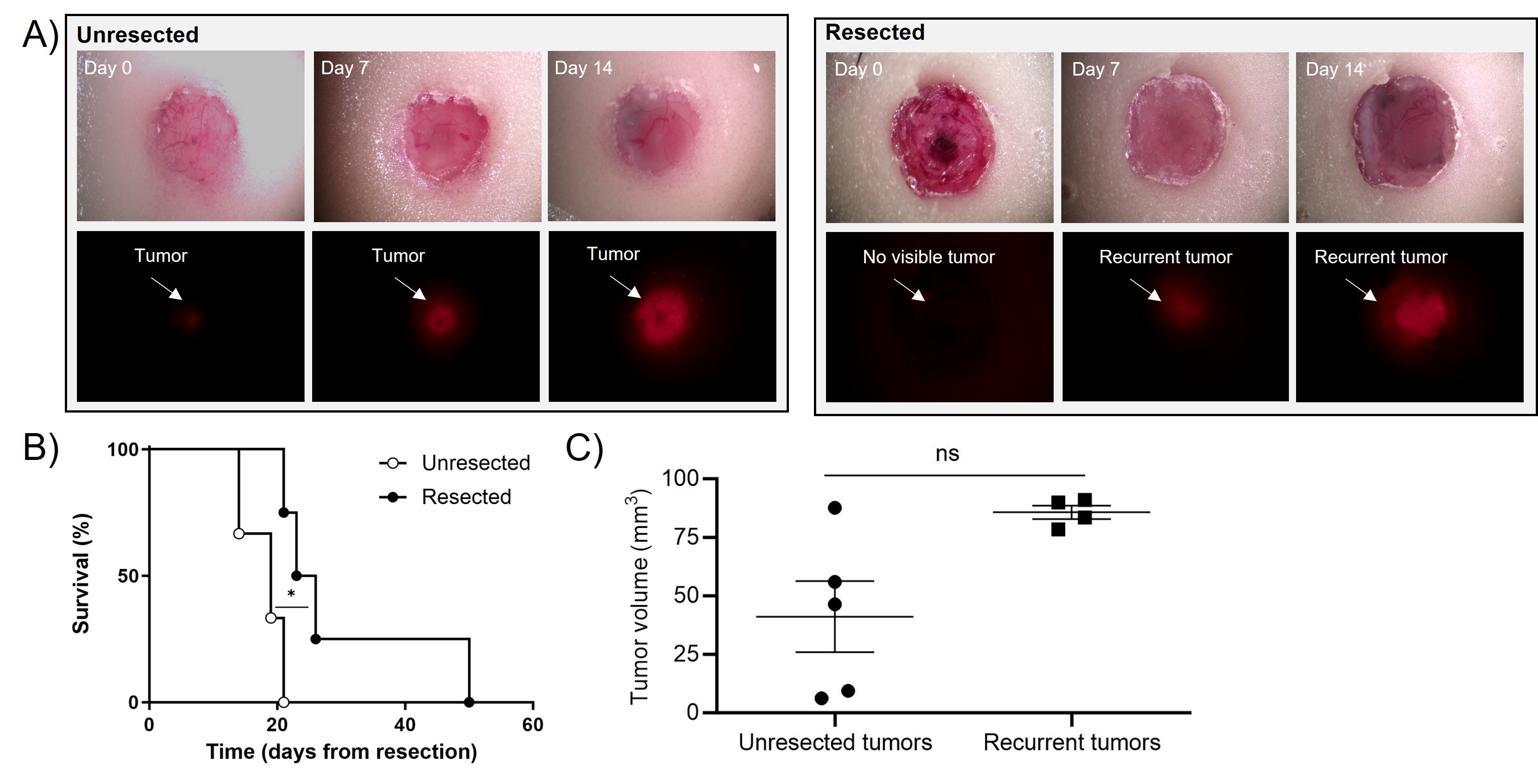


**Figure S1. Imaging of the brain microenvironment from surgery to recurrence** (**A**) Light microscope (upper panel) and fluorescent microscope (lower panel) images of transgenic mice bearing GL261-DsRed tumors and the cranial window in unresected (left panel) and resected animals (right panel). Images were taken at day 0 (immediately after the surgery and/or establishment of a cranial window) and day 7 and 14 post-surgery and/or cranial window implantation; (**B**) Kaplan-Meier survival curves of the unresected and resected transgenic mice bearing GL261-DsRed tumors (n=3-4; **p* < 0.05); (**C**) Quantification of the tumor volume of 3D light-sheet microscopy images of C57BL/6 mouse brains bearing GBM tumors 14 days post-surgery and/or cranial window implantation (mean ± SEM, n=4-5; not significant, unpaired Mann Whitney nonparametric test).


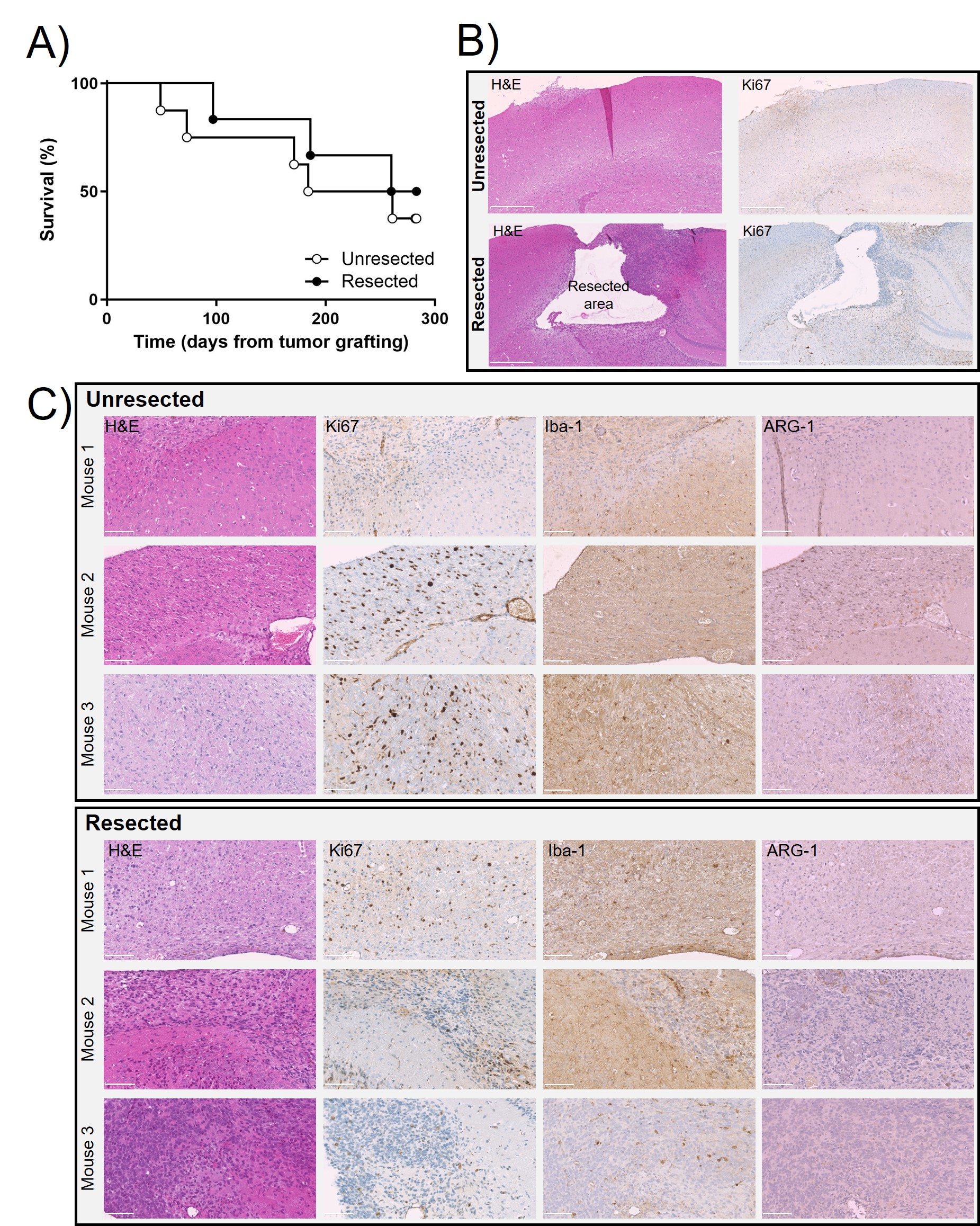


**Figure S2. Patient-derived GBM9 xenograft orthotopic resection model.** (**A**) Kaplan-Meier survival curves of the unresected and resected mice bearing GBM9 xenograft tumors (n=6-8); Median survival was 222.5 days for unresected animals and 271.5 for resected animals; (**B-C**) hematoxylin-eosin histology (B-C) and anti-Ki67 (B-C), Iba-1 (B) and ARG-1 (B) immunohistochemistry of three animals per group sacrificed nine months post-tumor grafting. Scale bar: 500 µm for B, 100 µm for C. A representative image with the presence of tumor cells is shown.


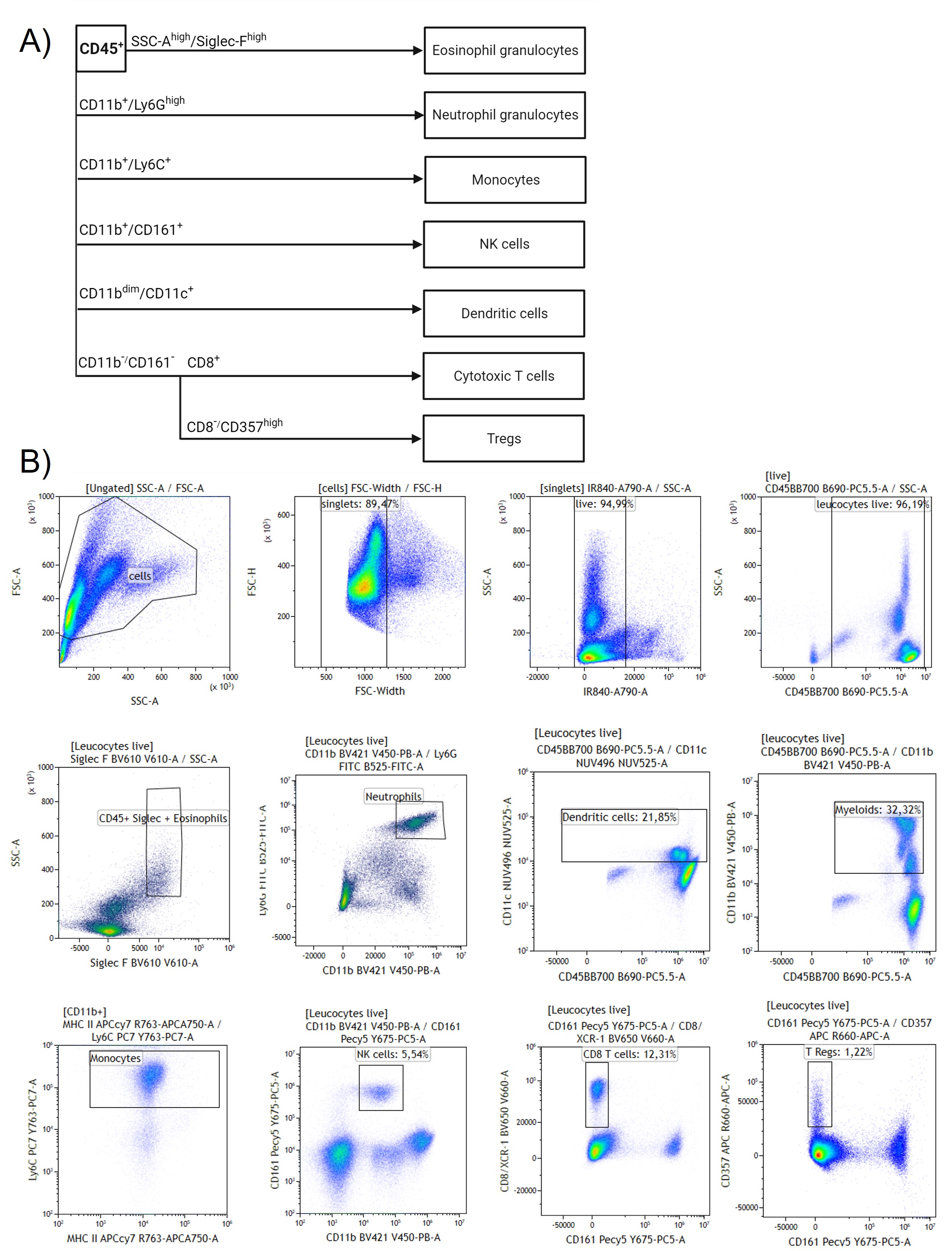


**Figure S3.** (**A**) Scheme of the gating strategy used for analyzing the white blood cells by multiparametric flow cytometry; (**B**) Flow cytometry gating strategy for blood samples analysis presented in Figure 1.

**
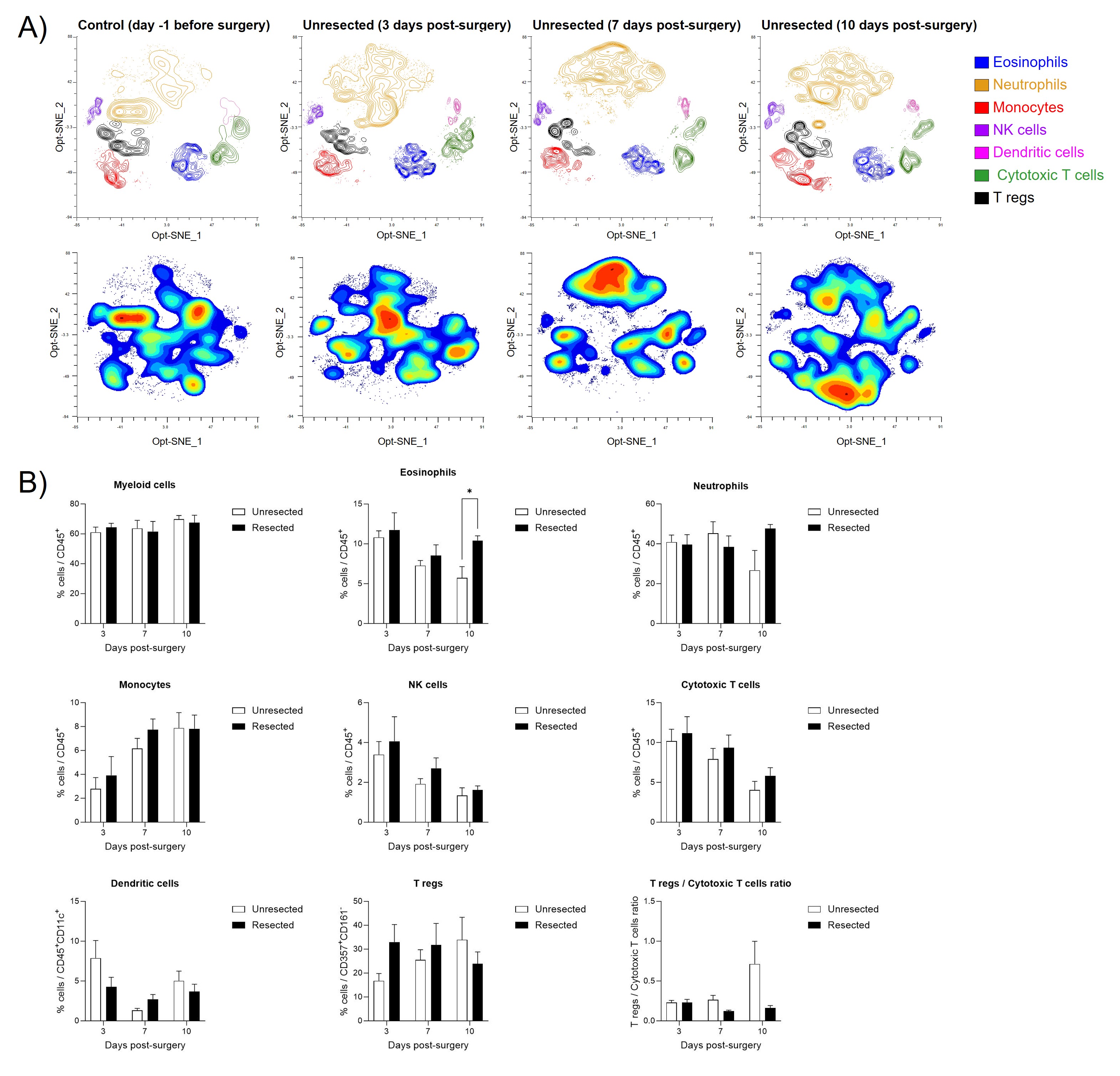
**

**Figure S4. Longitudinal characterization of the impact of surgery on tumor recurrence: mouse model and systemic immune modifications.** (**A**) Identification of immune cell population in mice blood using flow cytometry. The upper panels show Opt-SNE of manually selected cell cluster validated by FlowSOM analysis for control and unresected animals at day 3, 7 and 10 post-surgery. The lower panel represents, for the same groups of animals, the density contour plot of the different immune cell clusters in mice blood projected onto an Opt-SNE map; (**B**) Frequency of repartition of blood immune cell clusters for unresected and resected animals at day 3, 7 and 10 post-surgery. Numbers are expressed as percentage of cells related to CD45^+^ cells (for myeloid cells CD11b^+^, eosinophils, neutrophils, NK cells, Cytotoxic T cells), CD45^+^CD11c^+^ cells (for dendritic cells), CD357^+^CD161^-^ (for Tregs). The lowest right panel represents the ratio between Tregs and Cytotoxic T cells. (average ± SEM, n=5-9; Unpaired t test with Welch correction, **p*<0.05).


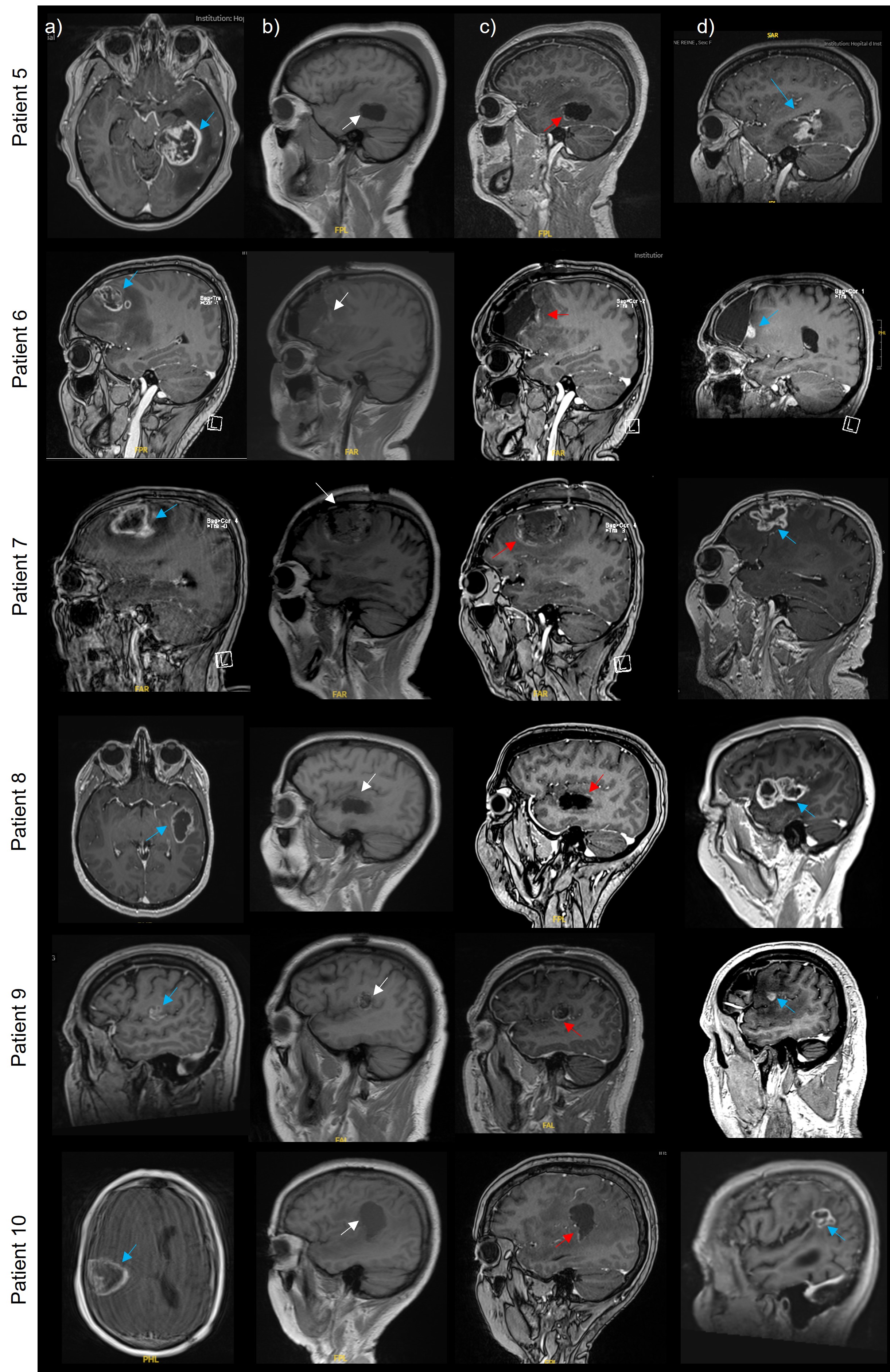


**Figure S5.** Illustrative brain MRI imaging of the remaining seven patients included in the study. The images show a T1 sequence with (a, c, d) or without (b) contrast enhancement, before (a) or after gross total resection (b and c) and at relapse (d). The blue arrows show the initial and recurrent tumor location, the white arrows show the tumor cavity location (b) and the red arrows show contrast enhancement associated with post-surgery BBB opening (c).


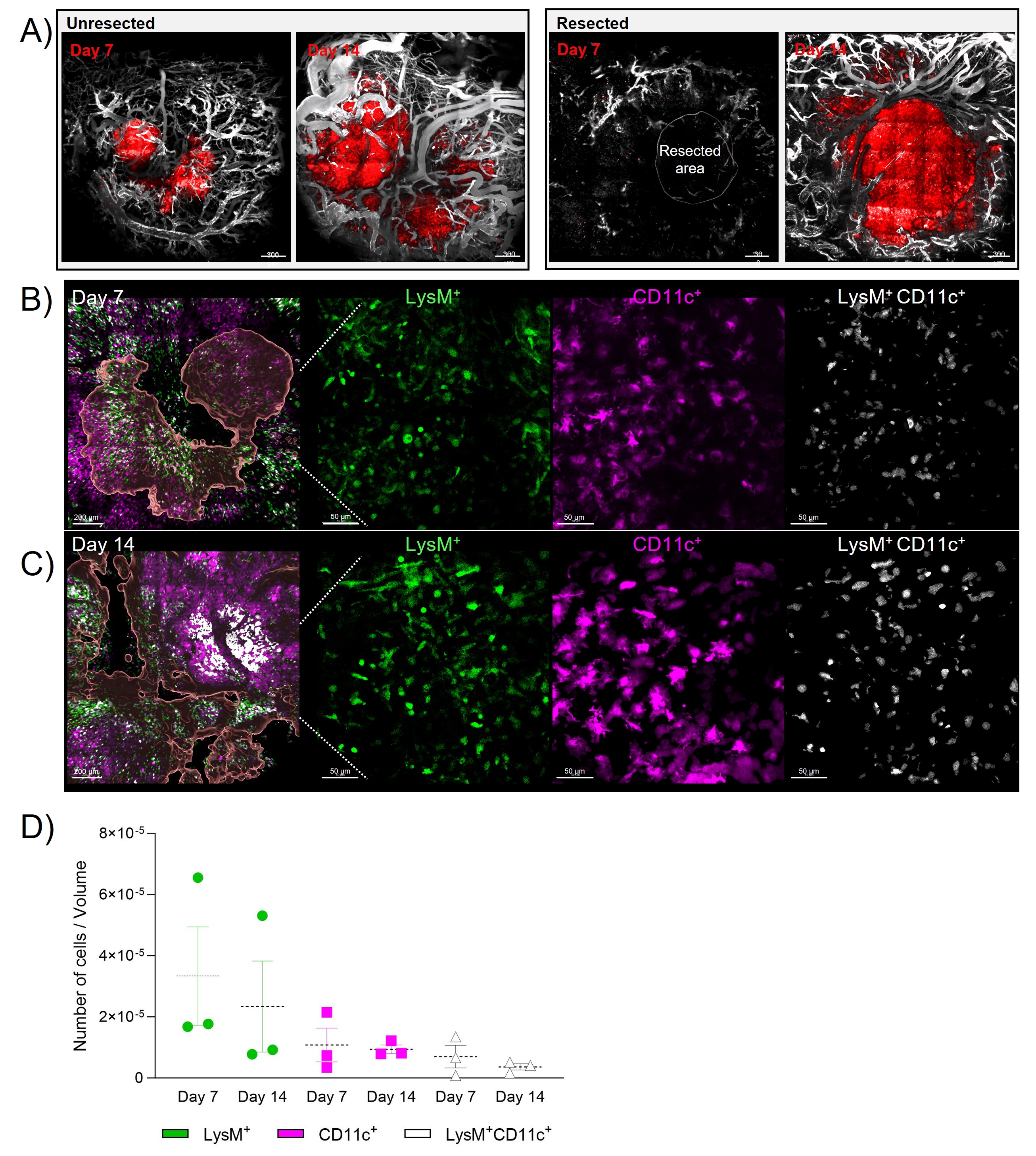


**Figure S6.** (**A**) Two-photon images at day 7 (left panels) and 14 post-implantation (right panels) of the cranial window (unresected) or resective surgery and cranial window implantation (resected). Tumors are represented in red, blood vessels in white (scale bar = 300 µm). In the left panel of the resected images, the resected area is represented as 3D reconstruction in white; (**B-C**) Two-photon images at day 7 (panel B) and 14 (panel C) post-implantation of the cranial window showing LysM-EGFP^+^ cells (green), CD11c-EYFP^+^ cells (purple), LysM-EGFP^+^CD11c-EYFP^+^ cells (white). The reconstruction of the tumor is represented in red in the left panels. Scale bar = 200 µm in the left panels, 50 µm in the three right panels: (**D**) Quantification of the number of cells per volume of LysM^+^, CD11c^+^ and LysM^+^CD11c^+^ cells at day 7 and 14 in the resected animals. The bar graphs represent mean ± SEM (paired nonparametric Wilcoxon test, not significant difference between day 7 and 14)..


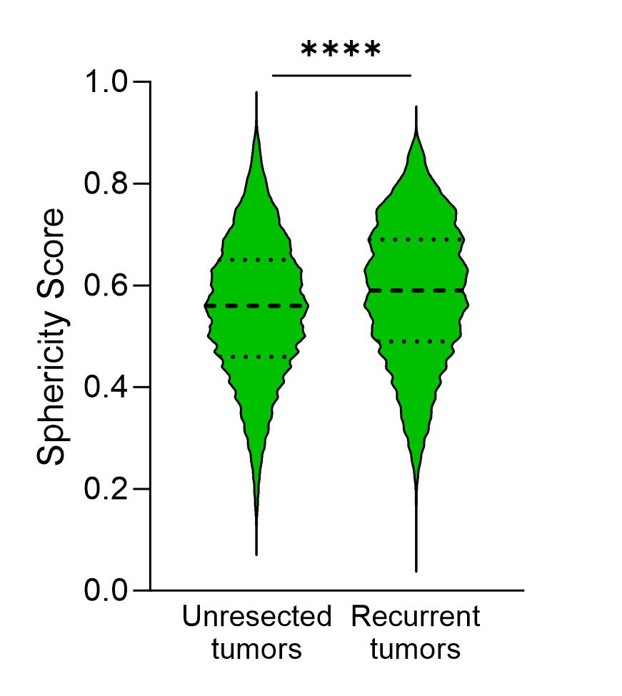


**Figure S7.** Sphericity score of TMEM119^+^ cells in 3D light-sheet microscopy images of brains of C57BL/6 mice bearing GBM tumors 14 days post-surgery and/or cranial window implantation (mean ± SEM, n=4-5; unpaired Mann Whitney nonparametric test, ****p<0.0001).


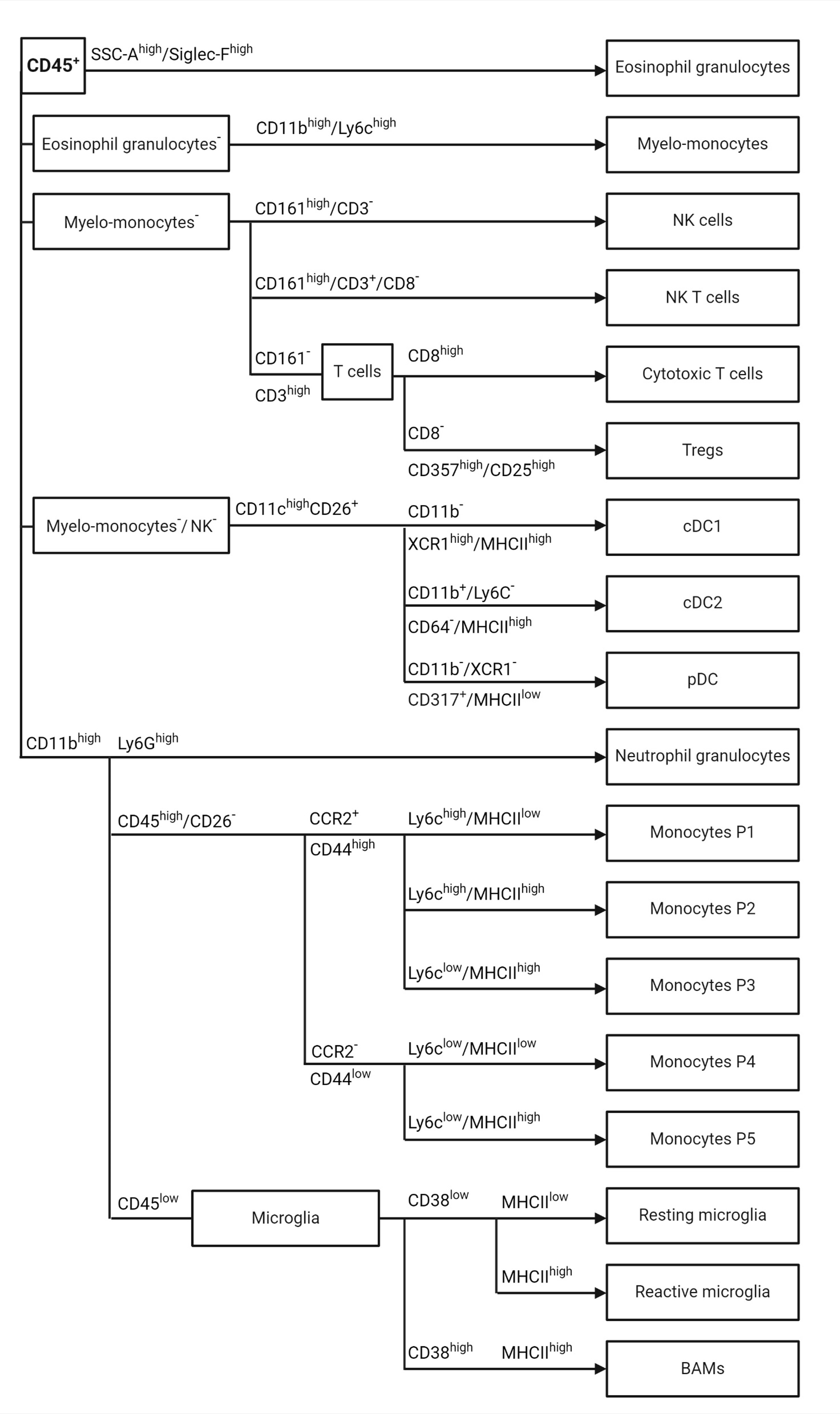


**Figure S8.** Scheme of the gating strategy used to analyze the tumor microenvironment in unresected and recurrent tumors by flow cytometry (DC: dendritic cells; BAM: border-associated macrophages).

**
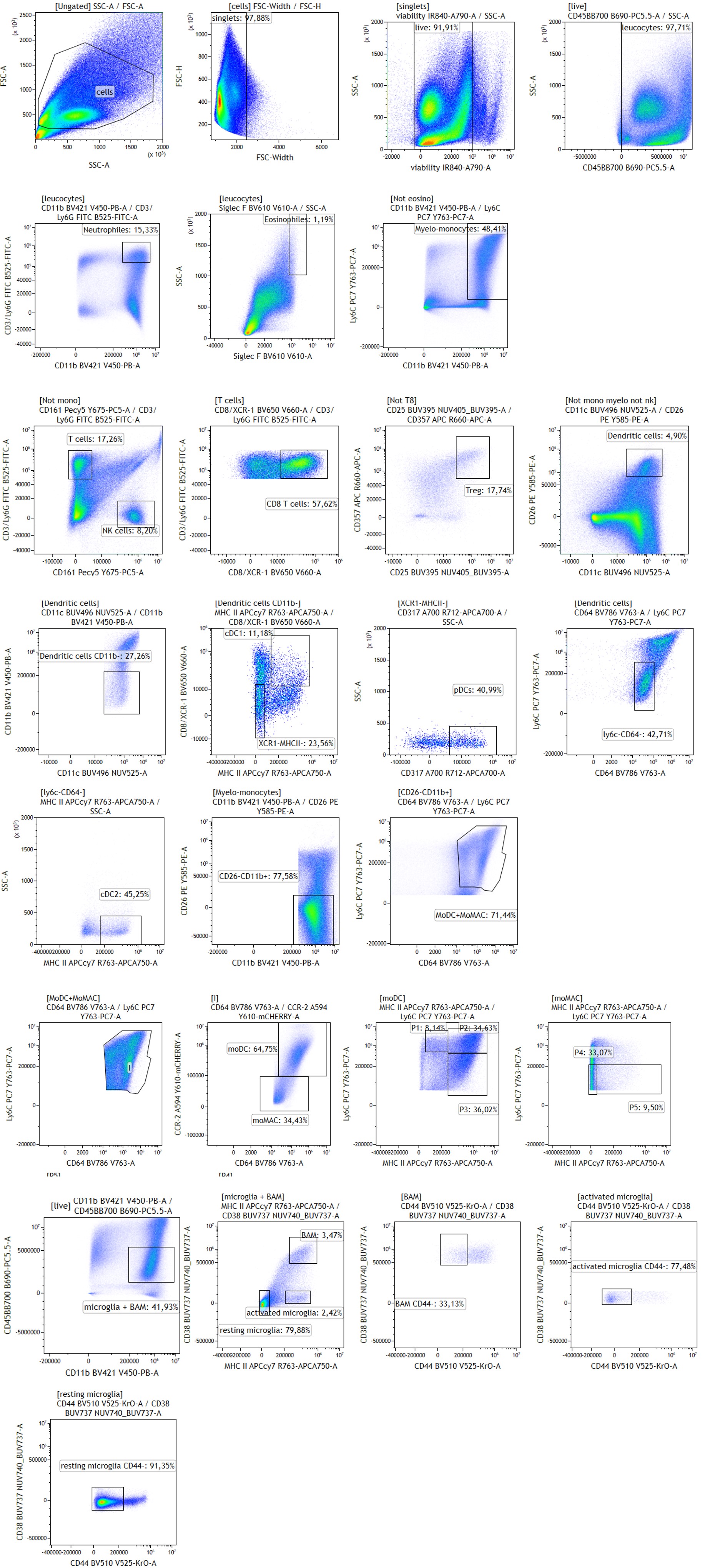
**

**Figure S9.** Flow cytometry gating strategy for brain tumor samples analysis.

**
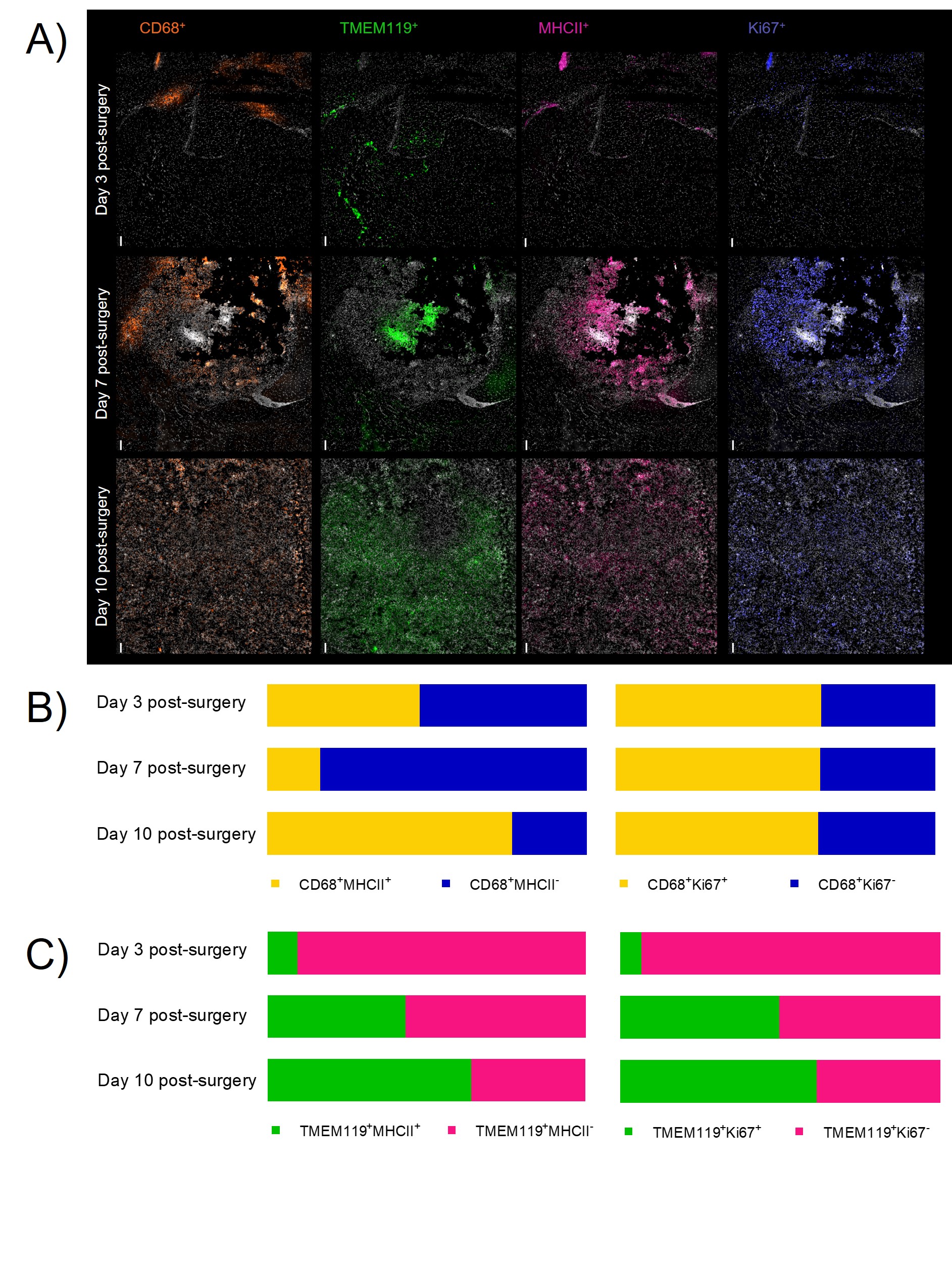
**

**Figure S10. The immune landscape of the SMe.** (**A**) Representative images of post surgical resection cavity in GL261DsRed bearing animals imaged with MACSima Technology during recurrence development at day 3, 7 and 10 post surgery stained with DAPI (white), CD68 (orange), TMEM119 (green), MHCII (magenta), Ki67 (blue). Scale bar: 100 µm; (**B-C**) Percent of MHCII^+^/MHCII^-^ (left panels) and as Ki67^+^ /Ki67^-^ (right panels) were quantified among the total CD68^+^ (panel **B**) and the total TMEM119^+^ (panel **C**) cells in the region of interest of hyperplexed immunofluorescence images during GL261 recurrence development at day 3, 7, 10 post-surgery (average value from 3 different slices).
